# Comparative Profiling of Mouse and Human Microglial Small Extracellular Vesicles Reveals Conserved Core Functions with Distinct miRNA Signatures

**DOI:** 10.3390/cells15020184

**Published:** 2026-01-19

**Authors:** Amir-Hossein Bayat, Damien D. Pearse, Praveen Kumar Singh, Mousumi Ghosh

**Affiliations:** 1 The Miami Project to Cure Paralysis, University of Miami Miller School of Medicine, Miami, FL 33136, USA; axm8710@med.miami.edu (A.-H.B.); dpearse@med.miami.edu (D.D.P.); pks72@med.miami.edu (P.K.S.); 2 The Department of Neurological Surgery, University of Miami Miller School of Medicine, Miami, FL 33136, USA; 3 The Neuroscience Program, University of Miami Miller School of Medicine, Miami, FL 33136, USA; 4 The Interdisciplinary Stem Cell Institute, University of Miami Miller School of Medicine, Miami, FL 33136, USA

**Keywords:** microglia, small extracellular vesicles, sEVs, BV2, HMC3, neuroprotection, human Schwann cells, miRNA

## Abstract

**Highlights:**

**What are the main findings?**

- Mouse and human microglial cell lines secrete small extracellular vesicles that exert shared core functional effects on human Schwann cells while displaying distinct molecular signatures.
- Cross-species miRNA profiling identifies a large, shared miRNA core with distinct, species-biased enrichment patterns.

**What are the implications of the main findings?**

- Divergent microglial sEV cargo between species highlights limitations when extrapolating rodent sEV data to human biology.
- This study provides a controlled cross-species framework for interpreting microglia-derived sEV function in human-relevant systems.

**Abstract:**

Microglia-derived small extracellular vesicles (MGEVs) are key mediators of neuroimmune communication, yet their cross-species comparability and translational relevance remain poorly defined. Here, we establish a harmonized framework to compare the molecular and biochemical signatures of sEVs derived from immortalized mouse (BV2) and human (HMC3) microglial cells as well as assess their bioactivity on a human Schwann cell (HuSC) line. MGEVs were isolated via MISEV-aligned size-exclusion chromatography (SEC) and characterized by nanoparticle tracking analysis (NTA), transmission electron microscopy (TEM), and immunoblotting for canonical EV markers CD9, CD63, CD81, TSG101. Human and mouse MGEVs exhibited similar morphology but displayed distinct membrane tetraspanin protein enrichment patterns. Functionally, mouse and human MGEVs attenuated HuSC migration while enhancing HuSC proliferation and their resistance to H_2_O_2_-induced oxidative stress, with human MGEVs providing stronger protective effects, suggesting they retain similar core functional properties. Short, non-coding-miRNA sequencing analysis identified 196 shared miRNAs (Spearman ρ = 0.72) with species-specific enrichment: human MGEVs-derived miRNAs favored regenerative and metabolic pathways, whereas mouse MGEVs-derived miRNAs aligned more so with inflammatory signaling. This study delivers the first integrated cross-species blueprint of MGEVs, revealing conserved neuroprotective actions alongside species-biased miRNA cargo that define translational boundaries and highlight human-relevant MGEV signatures for therapeutic innovation, therefore contributing to the importance of considering these differences in translational research.

## 1. Introduction

Microglia are the resident macrophages of the central nervous system (CNS) and are crucial for tissue homeostasis, immune surveillance, and responses to injury [1,2,3]. In the healthy brain, microglia constantly extend and retract their processes to monitor the parenchyma, a dynamic behavior first visualized in vivo using two-photon microscopy [4]. When challenged by pathogens, trauma, or neurodegeneration, microglia adopt context-dependent functional states that influence neuronal survival, synaptic remodeling, and repair [5].

Among the mechanisms that enable microglia to coordinate brain responses, extracellular vesicles (EVs) have emerged as key mediators of intercellular communication [6,7]. EVs are lipid bilayer-enclosed particles released by most cell types and comprise heterogeneous populations. Because EV isolates typically contain vesicles generated via multiple pathways, they should not be considered synonymous with exosomes [8]. Importantly, exosomes and microvesicles/ectosomes are defined by biogenesis: exosomes originate as intraluminal vesicles within multi-vesicular bodies and are released upon MVB–plasma membrane fusion, whereas microvesicles/ectosomes arise by outward budding from the plasma membrane [9,10]. In contrast, small/large EV terminology is size-based; therefore, consistent with ISEV recommendations, we use operational nomenclature and refer to them as small EVs (<200 nm) and medium/large EVs (>200 nm) [11,12]. sEVs, which originate from the endosomal pathway, carry canonical proteins which include tetraspanins CD9, CD63, CD81, and ESCRT components such as TSG101/ALIX, in addition to a range of parent-cell-specific proteins, lipids, and nucleic acids that reflect the phenotypic state of the EV-producing donor cell [9,13]. The International Society for Extracellular Vesicles (ISEV) recently updated community guidelines (MISEV2023) that standardize sEV isolation, characterization, and reporting, which we have followed in this work [12].

Microglia-derived sEVs (MGEVs) influence CNS biology in both protective and pathogenic ways [7,14,15]. In regenerative contexts, MGEVs can promote oligodendrogenesis, white-matter repair, and functional recovery, in part via sEV-carried microRNAs such as miR-23a-5p [16,17]. Conversely, under inflammatory conditions, MGEV cargo can propagate synaptic dysfunction and neurotoxicity [5,18,19]. Recent thematic reviews have addressed this duality and have highlighted the therapeutic potential of MGEVs across neurological disorders, including CNS injury and neurodegenerative diseases [20,21,22,23,24,25].

Accumulating transcriptomic and functional studies indicate that although mouse and human microglia share core homeostatic features, they differ substantially in immune signaling, metabolic programs, and receptor expression, raising important considerations for cross-species translation of microglial biology [26,27,28,29,30]. Accordingly, despite the widespread reliance on murine models to investigate MGEV function, growing evidence demonstrates marked transcriptional and epigenetic differences between mouse and human microglia that differentially shape innate immune pathways, including type I interferon signaling, TLR-mediated sensing, inflammasome activation, and NF-κB responsiveness [26,31]. Human microglia also display distinct lipid metabolic signatures and cholesterol-handling pathways, which influence vesicle biogenesis, membrane composition, and EV cargo loading. Furthermore, differences in receptor repertoires, including chemokine, purinergic, and scavenger receptors, shape species-specific responses to injury, inflammation, and environmental cues. These apparent cross-species disparities suggest that mouse microglia may produce sEVs with molecular profiles and functional outputs that do not fully recapitulate those of human microglia, although interpretation of such differences must also consider the influence of in vitro culture conditions and cell model-specific factors. These species-dependent differences make it difficult to extrapolate rodent MGEV findings to human biology, emphasizing the need for direct cross-species comparisons of vesicle composition and function.

This study employs immortalized microglial cell lines BV2 and HMC3 of mouse and human origin, respectively, which are widely used and retain many core microglial cell characteristics, enabling controlled and reproducible cross-species comparisons. While immortalization and prolonged in vitro adaptation may influence cellular metabolism, activation thresholds, and vesicle biogenesis, the use of these standardized models allows systematic identification of conserved and divergent sEV features across species. Accordingly, any observed differences in sEV composition or function may reflect both species-associated biology and model-specific effects, and the findings of this study are therefore intended to define comparative sEV signatures within controlled microglial systems rather than to directly replicate in vivo microglial cell behavior.

Here, we present the first integrated cross-species framework directly comparing sEVs from mice and human immortalized microglial cell lines produced under analogous culture conditions and employing the same sEV purification pipeline. For these investigations the use of mouse BV2 [32] and human HMC3 [20] microglia enable standardized, large-scale vesicle production and comparative studies. The sEV secretomes of these microglia have not been systematically compared to date under standardized and consistent conditions in the literature. Using MISEV-compliant isolation and enrichment [12], we couple biophysical and miRNA profiling with functional assays in a common recipient cell, human Schwann cells (HuSC). HuSC exhibit several common functional and phenotypic properties to microglia, notably overlapping molecular pathways associated with oxidative stress and the regenerative responses of microglia [33]. Further, we have recently explored the interaction of host innate immune cells with transplanted Schwann cells in models of spinal cord injury (SCI) [34] where sEVs likely play an important role in the intercellular communication between transplant and host. Together, this study addresses a critical gap in the field by providing the first controlled, side-by-side evaluation of conserved and species-specific MGEV features under matched conditions for vesicle production and purification. By establishing a standardized comparative platform, this work refines the translational interpretation of rodent MGEVstudies and extends beyond prior investigations that have largely focused on single species or disease-specific contexts [16,18,22].

## 2. Materials and Methods

### 2.1. Microglial and Schwan Cell Culturing

**Microglial cell culture.** The murine BV2 microglial cell line (derived from C57BL/6), widely used as a surrogate model for primary microglia [32,35], was maintained as previously described in our laboratory [36]. Human HMC3 microglial cells (ATCC^®^ CRL-3304™, Manassas, VA, USA; [20]) were cultured in tissue-culture-treated flasks (Thermo Fisher Scientific, Cat. # 130190, Waltham, MA, USA) using the same growth media and analogous culture conditions to BV2. In brief, microglia were grown in Dulbecco’s Modified Eagle Medium (DMEM, high glucose, Cat. # 11965092, Thermo Fisher Scientific, USA) supplemented with 10% heat-inactivated fetal bovine serum (FBS) (Cat. # A3840001, Thermo Fisher Scientific, USA) and 1X penicillin–streptomycin (Cat # 15140-122, Thermo Fisher Scientific, Waltham, MA, USA) [36]. Cultures were maintained at 37 °C in a humidified 5–7% CO_2_ atmosphere. To minimize activation during routine passaging, trypsinization was avoided; instead, cells were gently detached with sterile cell scrapers and triturated to obtain single-cell suspensions prior to reseeding.

**Human Schwann cell culture.** Immortalized human Schwann cells (HuSC; hTERT ipn02.3 2λ, ATCC, Cat. # CRL-3392, Manassas, VA, USA) were cultured in tissue-culture-treated flasks that were pre-coated with poly-L-lysine [PLL,10 μg/mL, 30–60 min, room temperature] (P8920-100ML, Sigma-Aldrich, St. Louis, MO, USA) in culture media constituting high-glucose DMEM (Cat. # 11965092, Thermo Fisher ScientificWaltham, MA, USA) supplemented with 10% heat-inactivated FBS and 100 U/mL penicillin, and 100 μg/mL streptomycin (Cat. # 15140122, Thermo Fisher Scientific, Waltham, MA, USA) at 37 °C in a humidified 5–7% CO_2_ incubator. Cultures were fed every 2–3 days and passaged at 70–85% confluence following Trypsin-EDTA (final concentration of 0.05–0.25%)-mediated dissociation from the flask and reseeding in appropriate plates for functional tests.

### 2.2. sEV Isolation

**sEV conditioning and harvest from murine and human microglia.** For sEV production from mouse and human microglial cell lines, cells were seeded into T175 tissue-culture flasks (Cat. # 130190, Thermo Fisher Scientific, USA) and expanded to near confluence (BV2: ~50–60%; HMC3: ~85–90%). Growth medium was aspirated and the monolayers for each cell type were rinsed once with sterile PBS (10010023, Thermo Fisher Scientific, Waltham, MA, USA). Cultures were switched to EV-production medium consisting of the same basal DMEM supplemented with exosome-depleted FBS (Cat. # A2720803, Thermo Fisher Scientific, USA) and 1× penicillin–streptomycin (Cat. # 15140-122, Thermo Fisher Scientific, Waltham, MA, USA). Serum-free conditioning was not used for generation of sEVs to preserve a resting or low-activation microglial phenotype while avoiding the confounding effects elicited by bovine EVs and protein nanoparticles. Cultures were incubated for 18–24 h to generate conditioned medium, which was then collected for sEV isolation. This time frame was chosen to balance vesicle yield with preservation of microglial viability and a low-activation state. Serum-free conditions were avoided due to the sensitivity of microglia to serum withdrawal, which can induce stress responses and alter vesicle biogenesis and cargo composition. Instead, exosome-depleted FBS was used to minimize bovine EV contamination while maintaining physiological support for microglial homeostasis.

**Isolation and Purification of MGEVs.** Isolation and purification of sEVs involved sequential low-speed centrifugation and filtration for removal of cells and debris, followed by ultrafiltration-based concentration and size-exclusion chromatography (SEC) as the primary vesicle separation method. Conditioned media were collected from mouse and human microglial cell cultures. For each batch of purification, 150 mL of condition media were first clarified by centrifugation at 1500× *g* for 10 min at 4 °C and subsequently passed through a Corning^®^ bottle-top vacuum filter (Cat. # CLS431153-12EA; Sigma-Aldrich, Germany) to remove cellular debris. The filtrate was then concentrated using a Centricon 70+ centrifugal ultrafiltration device with a 100 KDa molecular-weight cutoff (Cat. # UFC710008; Sigma-Aldrich, Germany) at 3000× *g* for 30 min at 4 °C. For size-exclusion chromatography (SEC), 1 mL of the concentrated medium was applied to a pre-rinsed qEV2/35 nm column (IZON Science, Christchurch, New Zealand) equilibrated according to the manufacturer’s instructions. The eluate was collected in a low-protein-binding microcentrifuge tube (Cat. # 90410, Thermo Fisher Scientific, USA) as sequential, equal-volume fractions using an Izon Automatic Fraction Collector (AFC) (IZON Science, New Zealand), and fractions 1 to 10 were subjected to further analysis. Collected fractions were kept at 4 °C until downstream assessment of EV purity, particle size, particle counts, and yield. For all experiments, a minimum of three independent sEV preparations were generated and analyzed for each of the two microglial cell lines.

### 2.3. Characterization of Isolated sEVs

In accordance with MISEV guidelines, sEV preparations were characterized using a multi-parameter approach including (i) enrichment of multiple canonical vesicular markers (CD9, CD63, CD81, and TSG101), (ii) exclusion of a cellular contamination marker (calnexin) and absence of the cytoskeletal protein β-actin in the EV-enriched SEC fractions (β-actin was detected only in the parental whole-cell lysates), (iii) fraction-resolved nanoparticle tracking analysis (NTA), and (iv) transmission electron microscopy (TEM) to assess vesicle morphology and size.

**Western Blot (WB) Characterization of Canonical sEV proteins.** Following sEV purification, the ten sequentially collected fractions were quantitatively evaluated for the total protein concentration in each of the fractions using the Micro BCA™ Protein Assay Kit (Cat. # 23235, Thermo Fisher Scientific, USA), according to the manufacturer’s instructions.

To assess the ratio of the canonical sEV marker distribution in sequentially collected fractions, all 10 fractions from each batch were first evaluated for purity by probing for the presence of characteristic sEV markers in conjunction with the absence of calnexin. In addition, EV-enriched SEC fractions (2–5) and parental microglial whole-cell lysates were probed for β-actin as a marker of potential cellular protein/cellular fragment carryover. Calnexin is an endoplasmic reticulum marker that serves as a negative control for purified sEVs when using immunoblot analysis as detailed below. For human- and mouse-derived purified MGEV preparations, calnexin immunoreactivity was consistently detected in fractions 6–10, indicating residual cellular membrane contamination in later SEC fractions. Consequently, later SEC fractions (≥6), including fraction 9, were excluded from downstream analyses due to the presence of contaminant markers and absence of sEV-specific proteins, consistent with non-vesicular content, and were restricted only to pooled fractions 2–5, which were enriched for multiple canonical sEV markers and devoid of detectable calnexin signal. For all subsequent analysis, sEV-purified fractions 2–5 were combined and concentrated using Amicon Ultra 100 kDa 4 mL centrifugal filters (Cat. # UFC810008, Millipore Sigma, St. Louis, MO, USA) at 1000× *g* for 5 min at 4 °C, to a final volume of ~1.5 mL. Parent microglial cell lysates (BV2 or HMC3, resting phenotype) were included in each gel as controls for probing for total cellular proteins.

For immunoblotting, samples were mixed 1:1 with 2× Laemmli buffer (Cat. #. 1610737EDU, Bio-Rad, Hercules, CA, USA) and heated for 10 min at 95 °C. For detection of the sEV-specific major tetraspanin membrane proteins (CD9, CD63, CD81), samples were analyzed under non-reducing conditions where β-mercaptoethanol (β -ME) was omitted to preserve disulfide-dependent epitopes according to previous reports by Kowal et al., 2017 [37]. TSG101 and calnexin, on the other hand, were run in the presence of reducing conditions. Equal amounts of protein were resolved by SDS PAGE using Mini-PROTEAN^®^ TGX™ 12% gels (Cat. # 4561045, or 4561046; Bio-Rad, USA) at 100–150 Volts for 60 min by Bio-Rad PowerPac™ Basic Power Supply (Cat. # 1645050, Bio-Rad, USA). Proteins were transferred to PVDF membranes (Cat. # 03010040001, Millipore, St. Louis, MO, USA) making use of PowerEase™ Touch 350W Power Supply (Cat # PSC350MB, Thermo Scientific, Waltham, MA, USA) with 20V for 60 min at room temperature. Then, membranes were blocked in 3% (*w*/*v*) BSA (Cat. # BAH62-0500, Equitech-Bio, Hamilton, OH, USA) in TBST [20 mM Tris-HCl (pH 7.5), 150 mM NaCl, 0.1% (*v*/*v*) Tween-20] for 1 h at room temperature and incubated overnight at 4 °C with each of the primary antibodies diluted to 1:1000 in blocking buffer: anti-CD9 (Cat. # sc-13118, Santa Cruz Biotechnology, Dallas, TX, USA; Cat. # 13403, Cell signaling technology, Danvers, MA, USA), anti-CD63 (Cat. # ab134045, Abcam, Waltham, MA, USA), anti-CD81 (Cat. # 10037, Cell Signaling technology, Danvers, MA, USA; Cat.#. 27855-1-AP, Proteintech, Rosemont, IL, USA), anti-TSG101 (Cat. # ab125011, Abcam, USA), and anti-calnexin (Cat. # ab133615, Abcam, USA). Anti-β-Actin (ACTB) Antibody (Catalog # A2228, Millipore Sigma, St. Louis, MO, USA). After three 5 min washes in TBST, membranes were incubated with appropriate HRP-conjugated secondary antibodies for 60 min at room temperature, followed by 3x TBST washes totaling 20 min. Signals were developed by chemiluminescence using SuperSignal™ West Pico PLUS (Cat. # 34580, Thermo Fisher Scientific, USA) and imaged on an Azure 600 system (Cat. # AZI600-01, Azure Biosystems, Dublin, CA, USA). Densitometry analysis was performed in ImageJ (v1.54g) with background subtraction applied uniformly across membranes. All immunoblotting was performed on a minimum of three independent MGEV batches per species, and marker profiles (CD63, CD9, CD81, TSG101 enrichment with concurrent calnexin depletion) were used to verify sEV-enriched fractions prior to downstream functional analyses. For all experiments, HuMGEVs and MsMGEVs were quantified following SEC purification by measuring total vesicle-associated protein count using the Micro BCA assay. sEV concentrations for in vitro assays were normalized based on protein content, and equal amounts of human- and mouse-derived MGEVs were used for all comparative assays. NTA was performed on each sEV preparation to verify particle size distributions and batch-to-batch consistency.

**Transmission Electron Microscopy (TEM).** Pooled sEV preparations (AFC fractions 2–5) from each experimental batch of the two cell types were concentrated with Amicon Ultra 100 kDa filters and prepared for TEM. Briefly, 20 microliter aliquots of each sample were applied to a carbon Formvar-coated copper grid for 30 min, rinsed in phosphate buffer, followed with fixing with aqueous uranyl acetate solution for 5 min. Next, it was treated with 2% glutaraldehyde with a subsequent rinsing with double-distilled water and stained with a 2% aqueous uranyl acetate solution for 5 min. Grid was kept overnight protected from light and was viewed at 80 kV in a JEOL JEM-1400 transmission electron microscope (Akishima, Tokyo, Japan) and images were captured with an AMT NanoSprint 15 digital camera (Woburn, MA, USA).

**Nanoparticle Tracking Analysis (NTA).** To determine sEV size distribution and concentration, aliquots from each AFC fraction (1–10) were diluted in phosphate-buffered saline (PBS, pH 7.4) to a final volume of 1 mL and analyzed by nanoparticle tracking analysis (NTA) using NanoSight NS300 (Malvern Panalytical, Malvern, UK) at room temperature. Based on prior optimization, additional on-instrument dilution was performed immediately before infusion to achieve an acquisition density of 30–60 particles per frame. For each fraction, five consecutive 30s videos were recorded with the camera level set between 10 and 11 and a syringe-pump infusion rate of 30 µL/s; all instrument settings were held constant within an experiment. Videos were processed using NanoSight NTA software (v3.4) to obtain particle size distributions and concentrations. A minimum of three independent sEV batches were evaluated for each species.

### 2.4. EV Uptake and Functional Effects

**Comparative Uptake of Human vs. Mouse Microglia-Derived sEVs by Human Schwann Cells.** Parent human (HMC3) and mouse (BV2) microglial cells stably expressing an sEV reporter were generated by transduction with a lentiviral vector encoding a CD63-tdTomato fusion (Lentifect™ Purified Lentiviral Particles, Cat. # LP772-025, Genecopoeia, Rockville, MD, USA) and cultured to ~90% confluence. CD63-tdTomato^+^ sEVs were then purified from the conditioned media using the same protocol described above. HuSCs (5 × 10^3^ cells/well) were plated on PLL-coated glass chamber slides (Nunc Lab-Tek II CC2 Chamber Slide System, Cat. # 154941, Thermo Scientific, Waltham, MA, USA), grown to 60–80% confluence, and treated with CD63-tdTomato-labeled HuMGEVs or MsMGEVs at a final concentration of 10 µg/mL (total sEV protein), with equal amounts applied for each species and time point for 24, 48, or 72 h at 37 °C; medium-only cultures served as controls. After incubation, HuSCs were washed twice with PBS, briefly exposed to 0.1% Triton X-100 in 1× PBS (1–2 min) to remove surface-bound sEVs, and fixed with 4% paraformaldehyde for 30 min at room temperature. Schwann cell bodies and nuclei were counterstained with Alexa Fluor™ 488 Phalloidin (1:400; Cat. # A12379, Thermo Fisher Scientific, USA) and Hoechst (1:1000; Cat. # H3570, Invitrogen, Carlsbad, CA, USA), respectively. Confocal z-stack images were acquired using a Dragonfly 200 high-performance spinning disk confocal microscope (Andor Technology, Oxford Instruments, Concord, MA, USA) with constant acquisition settings. sEV uptake quantified in ImageJ/Fiji as background-subtracted tdTomato intensity per cell (or “uptake index” normalized to cell count). Due to intracellular trafficking of sEVs through the endosomal–lysosomal pathway, internalized CD63-tdTomato positive signals appear as discrete intracellular puncta representing accumulations of multiple vesicles within endosomal compartments rather than individual sEVs resolved at single-particle level. Experiments were performed in three biological replicates.

**Comparative evaluation of mouse and human microglial sEVs effect on cell proliferation.** HuSCs were seeded at 10,000 cells/well in PLL-coated 12-well plates (three biological replicates per condition) and assigned to three groups: (1) untreated HuSCs control, (2) HuSCs + MsMGEVs, and (3) HuSCs + HuMGEVs (both used at 3 µg/well [16]). The HuSCs were exposed to sEVs post-plating (once the cells were adhered to the bottom of the plate) and incubated for 24 h. HuSCs were allowed to grow to a confluency of ~70% cells followed with fixing with 4% paraformaldehyde (30 min, room temperature). The fixed cells were permeabilized with 0.1% Triton X-100 (10 min, RT) and blocked in 3% BSA in PBS (60 min, room temperature). Proliferation was assessed by immunocytochemistry (ICC) using human-specific anti-Ki67 antibody (clone MIB-1, host-mouse, Cat. # M7240, Agilent Technologies, Santa Clara, CA USA); this was used at a dilution of 1:200 for 2 h at room temperature) followed with a goat anti-mouse Alexa 488 as a secondary antibody, nuclear counterstaining by Hoechst 33342 (1:1000) (Cat. # H3570, Invitrogen, USA), and Alexa Fluor™ 594 Phalloidin (1:400 dilution; Cat # A12381, ThermoFisher Scientific, USA) to mark the cell body. Images were acquired using an inverted fluorescent microscope (iX85, Olympus, Japan). Staining intensity was measured with ImageJ (v1.54g) and normalized to nuclei count to determine the proliferation index. HuSCs were treated with HuMGEVs or MsMGEVs at an equivalent dose of 3 µg sEV protein per well, normalized across species. Representative high magnification images for each group were acquired using the Dragonfly spinning disk confocal microscope (Andor Technology, Oxford Instruments, Concord, MA, USA).

**Comparison of MsMGEV and HuMGEV Cytoprotective Effects.** A comparative assessment was performed to evaluate the protective effects of mouse and human MGEVs on cell viability and cell death under hydrogen peroxide (H_2_O_2_)-induced oxidative stress. Two sets of HuSCs (10,000 cells/well) were seeded on PLL-coated 48-well plates and grown to ~70% confluence. Purified human or mouse MGEVs (3 µg/well) were added and incubated for 24 h. Four groups were evaluated: control, H_2_O_2_, H_2_O_2_ + MsMGEV, and H_2_O_2_ + HuMGEV; these had five replicates each, except for the blank control (three wells). Control and H_2_O_2_ groups received culture medium instead of MGEVs. One set was subjected to the evaluation of cell viability using the CCK-8 assay while the second set was subjected to determining the comparative neuroprotective effects of mouse versus human MGEVs as outlined below.

**Human Schwann cell viability.** HuSC viability was measured with the Cell Counting Kit-8 [CCK-8] (Cat. # GK10001, GLPBIO, Montclair, NJ, USA) following the manufacturer’s protocol. HuSCs were rinsed once with PBS and exposed to 50 µM of H_2_O_2_ for 60 min to induce oxidative stress. A blank control (culture medium plus CCK-8 reagent without cells) was included to correct for background absorbance. Purified HuMGEVs or MsMGEVs were added at an equal dose of 3 µg sEV protein/well for both species. After treatment, HuSCs were incubated with 10% (*v*/*v*) CCK-8 for 120 min at 37 °C, and absorbance at 450 nm was recorded using a SpectraMax iD5e microplate reader (Molecular Devices, San Jose, CA, USA). Cell viability (%) was calculated using the manufacturer’s formula (GLPBIO.com).

$$Relative\;Viability(\%)=\frac{\left[A450nm\left(sample\right)-A450nm(blank)\right]}{\left[A450nm\left(control\right)-A450nm(blank)\right]}\times 100$$

**Human Schwann cell death.** To assess MGEV protection under H_2_O_2_-induced oxidative stress, HuSC exposed to H_2_O_2_ were treated with or without HuMGEV or MsMGEV as above (3 µg sEV protein/well) and stained with Hoechst 33342 (1 µg/mL; 1:1000; Invitrogen H3570) and propidium iodide (1 µg/mL; 1:200; Invitrogen P3566) for 10–20 min at room temperature. After two PBS washes, images were acquired on an inverted fluorescence microscope (X85, Olympus, Japan). ImageJ v1.54g was used to count total nuclei (Hoechst) and PI-positive nuclei.

$$Cell\;Death\left(\%\right)=\frac{PI-positive\;cells}{Hoechst-stained\;cells}\times 100$$

**Human Schwann cell migration**. A wound scratch assay was conducted to evaluate and compare the effects of mouse and human MGEVs on HuSC migration. HuSCs were seeded in 12-well plates and incubated overnight in complete medium (control, HuMGEV, and MsMGEV groups; *n* = 4 wells/group). At 90–95% confluence, a linear scratch was made in each monolayer using a sterile 200 µL pipette tip. Detached cells were removed by a gentle PBS rinse, and wells were replenished with fresh culture medium containing 10 µg/mL of each of HuMGEVs or MsMGEVs; control wells received medium only. Phase-contrast images were acquired at 0, 24, 48, and 72 h post-scratch at 10× magnification under standard culture conditions (37 °C, 5% CO_2_). Wound closure over time was quantified as a measure of cell migration using the Wound Healing Size Tool plugin of Image J.

### 2.5. miRNA Profiling and Bioinformatic Analysis

**miRNA profiling of MGEVs by next-generation sequencing.** miRNA profiling of MGEVs was performed by System Biosciences (SBI, Palo Alto, CA, USA). Freshly collected MGEV-conditioned media were shipped to SBI on dry ice. Briefly, the SBI protocol involved first clarifying MGEV-containing conditioned media by centrifuging it at 3000× *g* for 15 min at 4 °C. The supernatant was carefully transferred to a fresh tube, and one-third of the total volume of ExoQuick™ TC reagent (Cat. # EXOTC10A-1, System Biosciences, SBI, USA) was added. The mixture was gently mixed by pipetting and incubated overnight at 4 °C. The following day, samples were centrifuged at 1500× *g* for 30 min at 4 °C, the supernatant was discarded, and the MGEV-containing pellet was retained for downstream RNA isolation.

Total RNA was extracted from MGEV preparations using SBI’s spin column-based RNA Purification Kit (Cat. # EQ806TC-1, System Biosciences (SBI), Palo Alto, CA, USA) according to the manufacturer’s instructions. RNA quantity and integrity, with particular attention to the small RNA fraction, were assessed on an Agilent 2100 Bioanalyzer (Santa Clara, CA, USA).

For small RNA library preparation, total MGEV RNA (minimum input 1 ng) was processed using the Qiagen small RNA library preparation kit. In practice, 5 μL of RNA per sample was used as input. Adaptor ligation was conducted using diluted adaptors (3′ adaptor diluted 1:5 and 5′ adaptor diluted 1:2.5 relative to the stock solutions), followed by reverse transcription and PCR amplification (22 PCR cycles). Successfully amplified libraries were subjected to gel extraction to isolate fragments corresponding to the expected small RNA insert size. Library yield and suitability for sequencing were confirmed by qPCR, and only libraries with a concentration > 1 nM were advanced to sequencing.

Indexed libraries were normalized and pooled to a final loading concentration of 1.45 pM without PhiX spike-in. Pooled libraries were sequenced on an Illumina NovaSeq X instrument (San Diego, CA, USA) using a paired-end 150 bp (PE150) run configuration, targeting an approximate depth of 10–15 million reads per sample. Raw sequencing data (FASTQ files) obtained from SBI via a secure web portal (SlugAnalysis, University of California, Santa Cruz, CA, USA) were subjected for subsequent bioinformatic analysis.

To minimize potential biases arising from library preparation efficacy, human and mouse MGEV RNA samples were processed using the same library preparation kit, protocol, input range, and sequencing platform to ensure maximal technical consistency. In addition, all samples were subjected to identical bioinformatic processing, normalization, and quality-control steps, reducing the likelihood that observed differences reflect technical rather than biological variation.

**Bioinformatic evaluation for screening for enriched and differentially expressed miRNAs (DE-miRNAs).** Small RNA sequencing data from human and mouse MGEV samples were processed with the nf-core/smrnaseq workflow (v2.3.0) [38], executed using Nextflow v23.04.1 [39] in a fully containerized Singularity environment to ensure reproducibility. Raw FASTQ files were initially assessed with FastQC v0.12.1 then subjected to adapter trimming, base-quality filtering, and low-complexity read removal using fastp v0.23.4. Species verification and small RNA composition profiling were performed with miRTrace v1.0.1 [40], which also filtered tRNA- and rRNA-derived fragments and confirmed the integrity of bona fide microRNA reads.

High-quality reads were aligned to precursor and mature miRNA reference sequences from miRBase release 22.1 using Bowtie v1.3.1 (INDEX_HAIRPIN/INDEX_MATURE; BOWTIE_MAP_HAIRPIN/BOWTIE_MAP_MATURE/BOWTIE_MAP_SEQCLUSTER). Alignment quality control, including mapping statistics, sorting, indexing, and coverage summaries, was conducted with Samtools v1.19.2. Quantification of mature and hairpin miRNAs and cluster annotation were performed using seqcluster v1.2.9 [41]. Reference FASTA/FASTQ formatting was handled with fastx_toolkit v0.0.14, and additional sequence parsing was performed with SeqKit v2.6.1. Final quantification outputs, including normalized counts-per-million (CPM) matrices and raw count tables, were generated in R v3.6.2 within the Nextflow execution environment. All workflow steps and intermediate files were automatically tracked by Nextflow, providing complete computational provenance.

All downstream statistical and visualization analyses were conducted in R v4.3.2. Quality assessment comprised principal component analysis (PCA), hierarchical clustering, and Spearman correlation heatmaps across biological replicates to verify sample grouping and identify potential outliers. CPM values were log_2_-transformed [log_2_(CPM + 1)] for exploratory visualization. For cross-species comparisons, human and mouse mature miRNA identifiers were harmonized by removing species prefixes (hsa-, mmu-) and intersecting shared miRNA species between datasets. Cross-species comparison was restricted to mature miRNAs with identical miRBase names (e.g., miR-146a-5p), which represent highly conserved miRNA families between human and mouse. Because these miRNAs share conserved seed sequences and regulatory functions, name-based harmonization is appropriate for assessing conserved vesicular miRNA biology. This approach specifically avoids including species-specific or poorly conserved miRNAs. Global human–mouse expression differences were evaluated by computing species-level mean CPM values and calculating log_2_(Human) − log_2_(Mouse) expression differences. Differences in abundance were not driven by sequencing depth or normalization artifacts, as both datasets were processed using identical trimming, mapping, and CPM normalization pipelines. These comparisons were visualized using scatterplots of shared miRNAs, rank–rank correlation plots, bar plots of enriched miRNAs, and heatmaps of the 50 most-divergent miRNAs, generated with ggplot2 v3.4.4 and pheatmap. These analyses correspond to sample correlation heatmaps, species-differential heatmaps, and enriched-miRNA bar plots.

Within-species differential expression analysis of mature miRNAs was performed using edgeR v3.42.4 [42]. Low-abundance miRNAs were removed using filterByExpr, and library sizes were normalized by the trimmed mean of M-values (TMM) method. A design matrix was specified according to sample group labels, followed by estimation of common, trended, and tagwise dispersions. Differential expression was tested using edgeR’s quasi-likelihood framework (glmQLFit followed by glmQLFTest), which provides improved control of type I error in small-sample settings [43]. Multiple testing correction was applied using the Benjamini–Hochberg procedure, with a false discovery rate (FDR) cutoff of ≤0.05. Quality-control visualizations, including multidimensional scaling (MDS) plots, biological coefficient of variation (BCV) plots, volcano plots, mean-difference (MD) plots, and heatmaps of the top differentially expressed miRNAs, were used to evaluate replicate consistency and dispersion structure. Differential expression result tables formatted with appropriate hsa- and mmu- prefixes were exported in a format suitable for downstream Ingenuity Pathway Analysis (IPA).

**Statistics**. Significant differences between groups were ascertained by a one-way analysis of variance (ANOVA) or a t-test, followed by a Bonferroni post hoc analysis using GraphPad Prism 10 (GraphPad Software, La Jolla, CA, USA). All data were evaluated using a α = 0.05 (95% confidence). The data shown in graphs are presented as the mean  ±  standard error of the mean (SEM), based on the number of replicates or independent experiments conducted. Asterisks included on the graphs indicate statistical differences between the treatment and control condition(s) with significance indicated at *** *p*  <  0.001, ** *p*  <  0.01, or * *p* <  0.05.

## 3. Results

To investigate species-specific properties of MGEVs (mouse and human) and their effects on recipient cells, we compared them using integrated biophysical, molecular, and functional analyses. We characterized SEC-isolated sEV fractions for physical properties, purity, protein markers, and ultrastructure; tracked fluorescently labeled vesicles in resting microglia of both species and their uptake kinetics in immortalized HuSC; and assessed species-specific effects on HuSC proliferation, migration, and resistance to oxidative stress. Finally, high-resolution miRNA sequencing and pathway enrichment analysis was conducted to identify conserved and divergent regulatory signatures, revealing shared and distinct biological features of resting-state mouse and human MGEVs.

**Figure 1:**
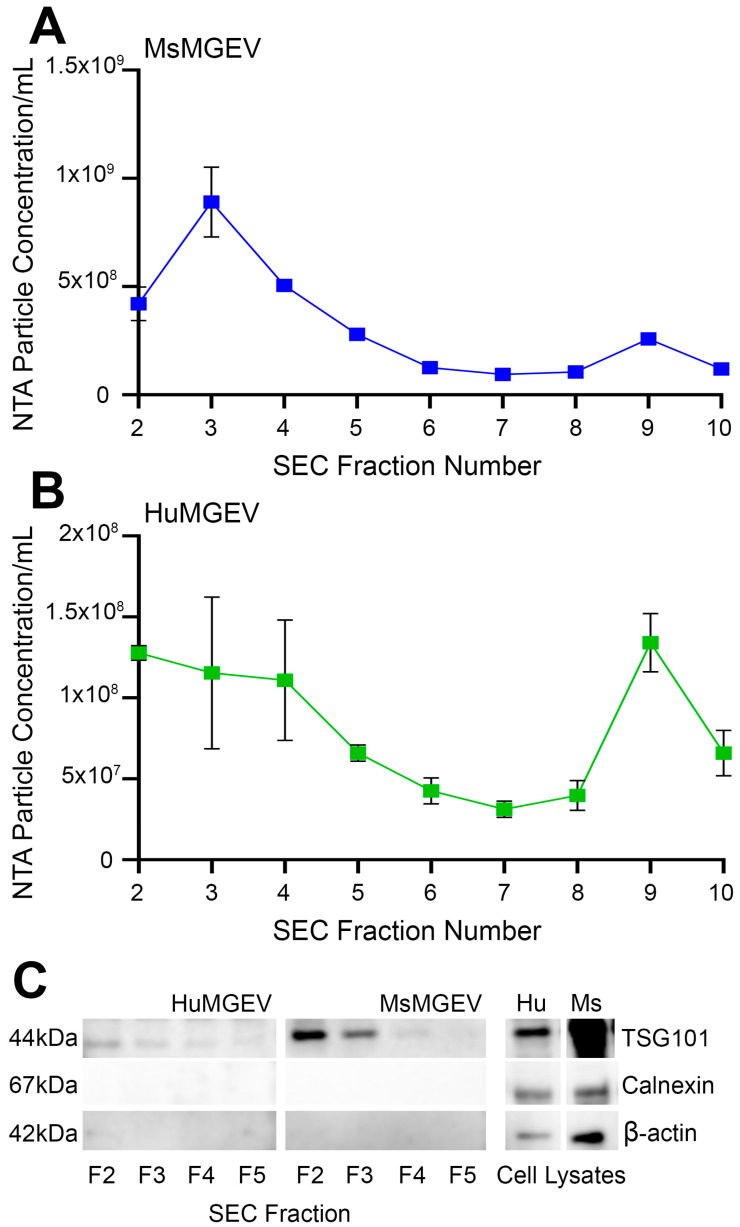
Characterization of size-exclusion chromatography-purified MsMGEV and HuMGEV fractions by nanoparticle tracking analysis and Western blotting. (**A**) Nanoparticle tracking analysis (NTA) of MsMGEVs collected from size-exclusion chromatography (SEC) fractions 2–10. Particle concentrations peaked in fraction #3, with subsequent fractions displaying progressively lower concentrations. (**B**) NTA of HuMGEVs from SEC fractions #2–10. Particle concentrations were highest in fractions 2–4, followed by a decline through fractions 5 thru7, and a secondary minor increase in fraction 9. Data represents mean values from three independent sEV purification batches. (**C**) Western blot analysis of SEC fractions #2–5 from HuMGEVs and MsMGEVs, probed for the sEV marker TSG101 (calculated molecular weight: ~44 kDa), the endoplasmic reticulum marker calnexin and a negative marker for sEVs (calculated/polypeptide molecular weight: ~67 kDa), and the total cell lysate marker β-actin (~42 kDa). TSG101 was enriched in SEC fractions that corresponded to the NTA-identified fractions with high particle concentrations, whereas calnexin was absent in these sEV-enriched fractions, indicating minimal cellular contamination. Representative blots of whole-microglial-cell lysates (Hu and Ms) show presence of both calnexin and β-actin. β-actin was detected in whole-cell lysates—but was absent from sEV-enriched SEC fractions (F2–F5)—together with calnexin, supporting low cellular contamination in the pooled sEV preparation.

**Biophysical and Molecular Characterization of Purified Microglia-Derived sEV Fractions.** A combination of sequential low-speed centrifugation followed with ultrafiltration and SEC were used to purify sEV-enriched fractions from mouse and human immortalized microglial cultures. NTA measurements of MsMGEV in the different fractions (#2–10) revealed a sharp peak of sEVs with respect to particle concentration in fraction #3, followed by a progressive reduction in the concentration in subsequent fractions, indicating that most vesicles eluted early during SEC separation (Figure 1A). In contrast, HuMGEV NTA profiles showed the highest particle abundance across fractions 2–4, with a gradual decrease through fractions 5–7, although a minor particle peak was observed in SEC fraction 9 of HuMGEV preparations (Figure 1B). As late SEC fractions were enriched in contaminating soluble cellular proteins rather than bona fide sEVs, this peak is unlikely to represent a purified vesicular population. Additionally, fraction 9 lacked enrichment of canonical sEV markers and coincided with detection of the cellular contamination marker calnexin. Accordingly, fraction 9 was excluded from all pooled sEV preparations and subsequent downstream analyses.

Western blot analysis of fractions 2–5 confirmed sEV enrichment, with TSG101 (~44 kDa) detected prominently in sEV-rich fractions from both species (Figure 1C). Calnexin (~67 kDa), an ER-associated negative marker for EV preparations, was not detected in SEC fractions 2–5. Similarly, β-actin (~42 kDa) was absent from these sEV-enriched fractions but clearly present in whole-cell lysates (Figure 1C), indicating minimal carryover of cellular proteins/fragments in the pooled fractions used for downstream experiments. Together with enrichment of canonical sEV markers, the lack of calnexin and β-actin in fractions 2–5 supports low contamination from cellular membranes/organelles and justified selective pooling of these fractions for all subsequent analyses [44,45,46,47]. Together, these data verify successful isolation of vesicle-enriched SEC fractions with minimal non-vesicular protein contamination supported by concordant SEC fraction profiling, enrichment of multiple canonical EV markers, exclusion of calnexin and β-actin, NTA assessments, and ultrastructural confirmation by TEM.

**Figure 2:**
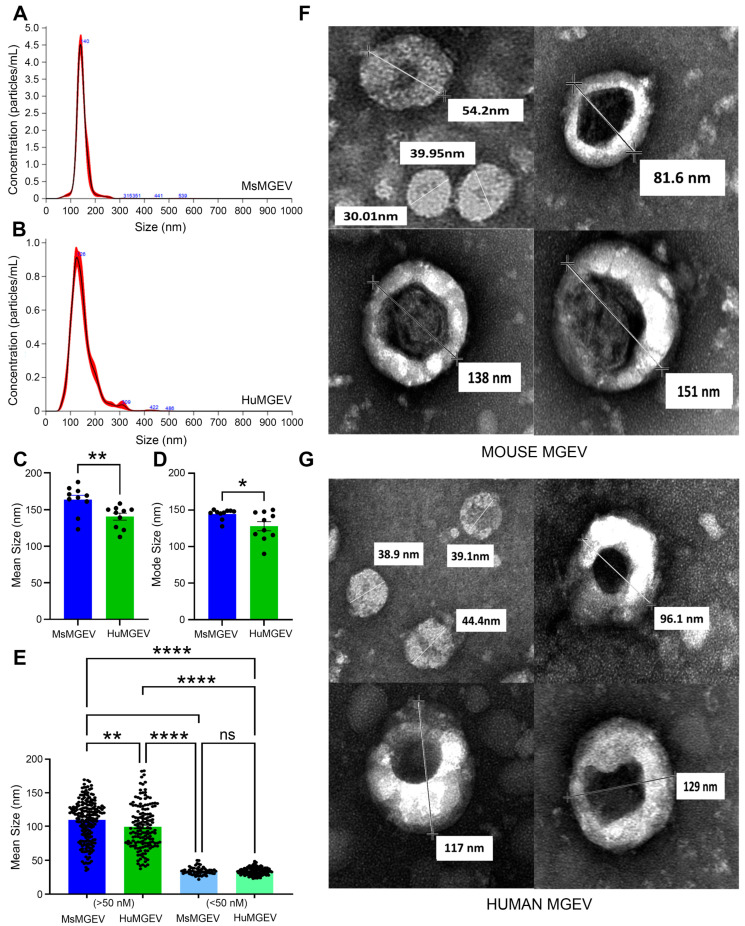
Comparative biophysical characterization and ultrastructural analysis of MsMGEVs versus. HuMGEVs. (**A**,**B**) NTA size distribution profiles of (**A**) MsMGEVs and (**B**) HuMGEVs. Representative traces from individual measurements are shown in red, with mean distribution profiles overlaid in black. Both mouse and human sEV exhibit a predominant peak between ~100–150 nm. (**C**,**D**) show quantitative analysis of sEV populations across both species, from multiple sEV+ fractions collected from independent purification batches; (**C**) mean [representing the full distribution including less abundant larger or smaller particles] and (**D**) mode [corresponding to the particle diameter of the most frequently occurring vesicle size within the measured population] particle sizes obtained from NTA measurements. MsMGEVs displayed significantly larger mean (** *p* < 0.01) and mode (* *p* < 0.05) diameters relative to HuMGEVs. Bars represent mean ± SEM; each point corresponds to an independent biological replicate. (**E**) Combined analysis of particle sizes from MsMGEVs and HuMGEVs measured by transmission electron microscopy (TEM). Both MsMGEVs and HuMGEVs exhibited two predominant sEV size populations by TEM (** *p* < 0.01, **** *p* < 0.0001): a larger group with diameters ranging from approximately 50–150 nm, and a smaller vesicle population measuring roughly 30–50 nm. Representative TEM images of negatively stained (**F**) mouse and (**G**) human MGEVs. Vesicles appear primarily spherical or donut-shaped with typical sEV morphology and electron-dense lumens. These images show bright halos characteristic of membrane-bound sEVs, arising from stain exclusion at the lipid bilayer and contrast between the vesicle lumen and surrounding stain, reflecting typical sEV ultrastructure. Representative vesicle diameters are indicated for each image panel, spanning ~30–150 nm across both species. Scale bars represent the magnification provided by the measurement overlays.

**Comparative Vesicle Size Distribution and Ultrastructure of MsMGEVs and HuMGEVs.** Biophysical profiling revealed clear species-associated differences in size distribution of MGEVs. Representative NTA traces measuring both mode [particle size referring to the most frequently occurring vesicle diameter reflecting the dominant sEV population] and the mean particle size, which is influenced by the full-size or range of distribution, showed that both MsMGEVs and HuMGEVs exhibited a dominant vesicle population between ~100–150 nm (Figure 2A,B). Quantitative analysis across multiple biological replicates demonstrated significantly larger mean (** *p* < 0.01) and mode (* *p* < 0.05) particle diameters in MsMGEVs compared to HuMGEVs, indicating intrinsic species-dependent size differences (Figure 2C,D). Transmission electron microscopy (TEM) demonstrated heterogeneity in vesicle populations for both species, revealing two primary size classes: a dominant group with diameters of approximately 50–150 nm, and a smaller subset ranging from roughly 30–50 nm (** *p* < 0.01, **** *p* < 0.0001) (Figure 2E). TEM images revealed characteristic sEV morphology, including spherical to donut-shaped structures with electron-dense lumens. Vesicle diameters within individual images spanned ~30–150 nm (Figure 2F,G). The ~30–50 nm vesicle subset was primarily resolved by TEM analysis, which offers higher spatial resolution and greater sensitivity than NTA for detecting small sEVs, and revealed two distinct vesicle size classes across species. These findings indicate that although MsMGEVs and HuMGEVs share overall ultrastructural characteristics, they differ significantly in their average particle size.

**Figure 3:**
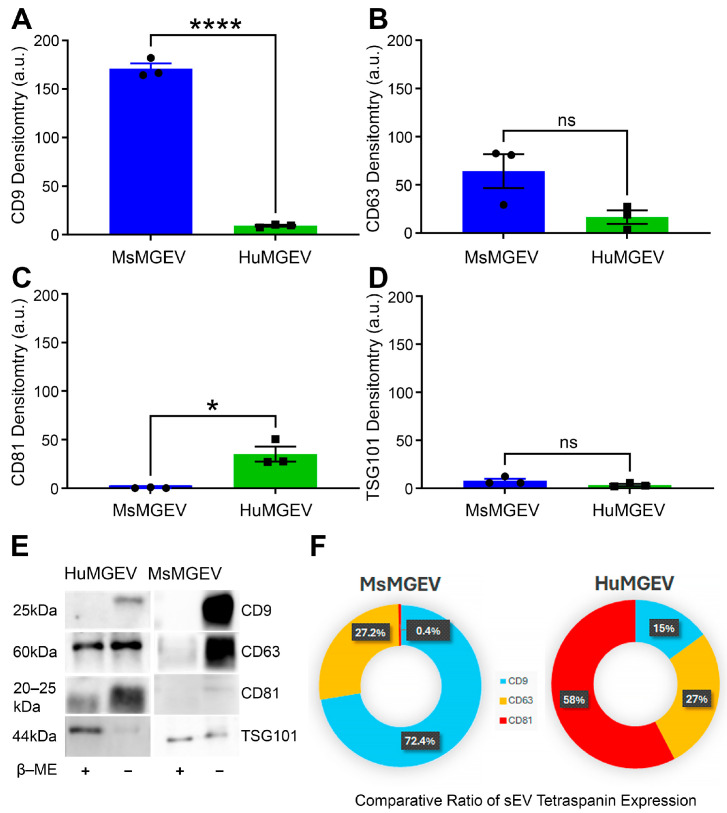
Comparative expression of canonical sEV markers in mouse and human microglia-derived sEVs. (**A**–**D**) Quantification of sEV membrane tetraspanin protein expression measured in MsMGEVs and HuMGEVs. Ratios represent protein concentration normalized band intensities from Western blots across three independent sEV preparations. (**A**) CD9 expression was markedly higher in MsMGEVs than in HuMGEVs (**** *p* < 0.0001). (**B**) CD63 levels showed no significant difference (ns) between species. (**C**) CD81 expression was significantly enriched in HuMGEVs relative to MsMGEVs (* *p* < 0.05). (**D**) TSG101 levels did not differ significantly across the two species (ns). Bars represent mean ± SEM, with individual data points shown. (**E**) Representative Western blots of sEV-enriched fractions from HuMGEVs and MsMGEVs, probed for CD9, CD63, CD81, and TSG101. Samples were analyzed under reducing (+β-ME) and non-reducing (**−**β-ME) conditions to assess tetraspanin multimerization states and epitope sensitivity. (**F**) Donut plots depict the percentage contribution of each tetraspanin (CD9, CD63, CD81) to the total tetraspanin signal, calculated from mean normalized band intensities across three independent biological experiments, illustrating species-specific differences in tetraspanin abundance between HuMGEVs (**right**) and MsMGEVs (**left**).

**Species-Specific Expression of Canonical sEV Membrane Proteins.** Western blot assessment of canonical sEV markers demonstrated distinct tetraspanin expression signatures between MsMGEVs and HuMGEVs. CD9 expression was markedly elevated in MsMGEVs relative to HuMGEVs (**** *p* < 0.0001) (Figure 3A), whereas CD63 levels were comparable across species (Figure 3B). Conversely, HuMGEVs displayed a significant enrichment of CD81 (* *p* < 0.05) (Figure 3C), highlighting species-dependent differences in tetraspanin incorporation into MGEVs. Because quantitative immunoblot signals can be influenced by species-dependent antibody affinities, all Western blot analyses were performed using validated human- or mouse-specific antibodies and quantified within species, with normalization to corresponding controls rather than direct cross-species band-intensity comparisons. TSG101 expression did not differ significantly between the two groups (Figure 3D) that may reflect a combination of an apparent species bias and cell line-specific regulatory programs. Representative blots demonstrated expected banding patterns under both reducing and non-reducing conditions (Figure 3E), revealing tetraspanin multimerization states and validating specificity of detection. Donut plot analyses further highlighted the unique proportional distribution of CD9, CD63, and CD81 within each species, highlighting the distinct molecular sEV fingerprints characteristic of mouse versus human MGEVs (Figure 3F).

**Figure 4:**
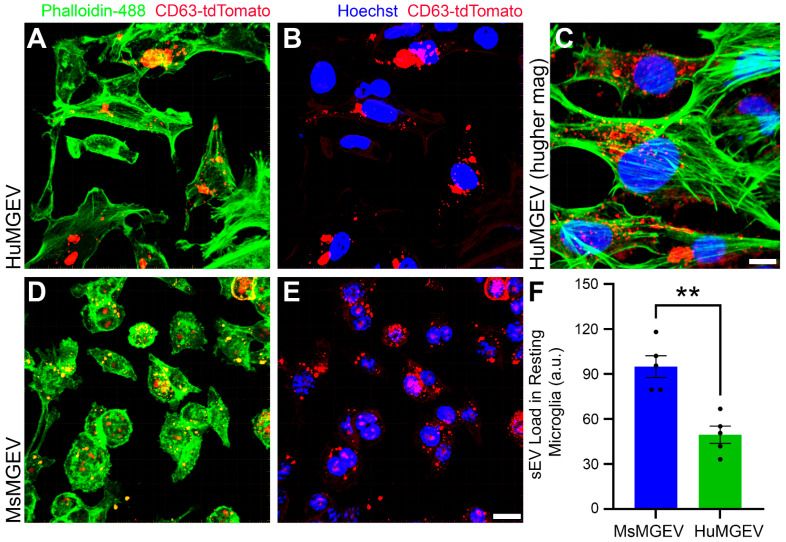
Robust expression exhibiting a comparative CD63-tdTomato-labeled vesicle load in resting microglia across species. (**A**–**C**) Confocal microscopy images of immortalized human microglia in resting state showing the presence of CD63-tdTomato-labeled vesicles (red). Cells were counterstained with Alexa Fluor™ 488 Phalloidin (green) to visualize microglial cell bodies and Hoechst (blue) to label nuclei. (**A**,**B**) Low-magnification view showing widespread intracellular distribution of CD63-tdTomato-labeled with majority exhibiting a perinuclear accumulation. Higher-magnification (40×) image of HuMG parent cells (**C**) demonstrating punctate CD63-positive vesicles localized along actin filaments and concentrated in the perinuclear region. (**D**,**E**) Resting mouse microglia exhibiting robust expression of CD63-tdTomato-labeled intracellular vesicles. (**D**) Phalloidin-488 staining defines cell boundaries and morphology, revealing widespread CD63-positive puncta throughout the cytoplasm. (**E**) Hoechst counterstaining confirms intracellular vesicle localization surrounding the nucleus. (**F**) Quantification of intracellular vesicle load in resting microglia of both mouse and human origin displayed significantly higher cellular load of CD63-expressing intracellular vesicles in microglia of mouse origin compared to that of human origin (** *p* < 0.01). Bars represent mean ± SEM; acquired from images obtained from independent biological replicates from each species. Scale bar: 15 µm (**A**,**B**,**D**,**E**) and 5 µm (**C**).

**Comparative Intracellular Vesicle Load in Resting Human and Mouse Microglia.** Confocal imaging of CD63- tdTomato-expressing microglia demonstrated robust intracellular vesicle labeling in both human and mouse cells under resting conditions (Figure 4A–E). In human microglia, tdTomato-labeled CD63-positive puncta were widely distributed throughout the cytoplasm with strong perinuclear accumulation, as visualized by co-staining with the cytoskeleton marker Phalloidin- 488 and nuclear labeling via Hoechst (Figure 4A,B). Higher-magnification imaging revealed vesicle clustering along actin filaments, consistent with trafficking through cytoskeletal networks (Figure 4C). Mouse microglia similarly exhibited widespread CD63-tdTomato-positive vesicles (Figure 4D,E); however, quantitative analysis revealed significantly higher intracellular vesicle load in mouse microglia compared with human microglia (** *p* < 0.01) (Figure 4F). These findings indicate species-dependent variation in basal vesicle abundance within microglial cells, with mouse cells exhibiting inherently greater intracellular vesicle content. Consistent with this observation, the increased intracellular vesicle burden in mouse microglia was accompanied by a higher overall yield of secreted sEVs compared with HuMGEVs, when quantified by NTA assessments (Figure 2A,B).

**Figure 5:**
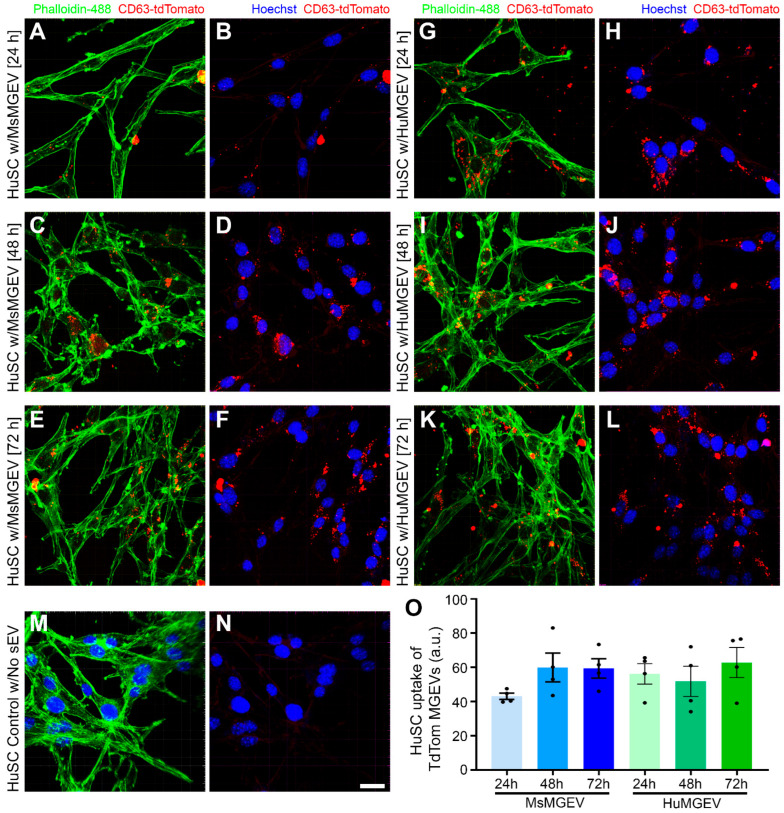
Time-dependent uptake of CD63-tdTomato-labeled MGEVs by HuSCs. (**A**–**F**) Confocal images of HuSC exposed to purified CD63-tdTomato-labeled MsMGEVs (red) for 24 h (**A**,**B**), 48 h (**C**,**D**), or 72 h (**E**,**F**). Cells were stained with Alexa Fluor™ 488 Phalloidin (green) to mark the cell body and Hoechst to label nuclei (blue). Internalized CD63-tdTomato-positive sEVs appear as red puncta distributed throughout the cytoplasm and concentrated in perinuclear regions. The size of fluorescent puncta reflects intracellular endosomal accumulation of multiple internalized sEVs rather than aggregation of individual vesicles. Uptake of MsMGEVs showed a trend in an increase in sEVs overtime. Confocal images of HuSC treated with purified CD63-tdTomato-labeled HuMGEVs, red) for 24 h (**G**,**H**), 48 h (**I**,**J**), or 72 h (**K**,**L**). Like MsMGEVs, HuMGEV uptake was evident as intracellular red puncta; however, the overall accumulation pattern remained relatively stable across time points. (**M**,**N**) shows untreated HuSC microglia (control with no MGEV exposure) showing phalloidin-488 and Hoechst staining but no detectable CD63-tdTomato signal, confirming that red puncta in treated groups represent internalized CD63-tdTomato-labeled MGEVs. (**O**) Quantification of CD63-tdTomato fluorescence intensity in HuSC cells following 24 h, 48 h, or 72 h exposure to MsMGEVs or HuMGEVs. Both vesicle types were taken up by HuSC microglia, with MsMGEVs showing a modest trend toward increased accumulation over time. Bars represent mean ± SEM; individual points represent biological replicates. Scale Bar: 15 µm.

**Time-Dependent Internalization of MsMGEVs and HuMGEVs by Human Schwann Cells.** HuSCs readily internalized CD63-tdTomato-labeled MGEVs, which appeared as enlarged intracellular red puncta distributed throughout the cytoplasm, consistent with endosomal accumulation of multiple internalized sEVs rather than individual fluorescent vesicles, which are not distinguishable at the resolution of confocal microscopy. Uptake dynamics appeared to differ modestly between MsMGEVs and HuMGEVs (Figure 5A–L,O). Following exposure to MsMGEVs for 24, 48, and 72 h, confocal imaging revealed increased intracellular puncta within the first 24 h (Figure 5A–F); however, quantitative fluorescence analysis showed no statistically significant differences in uptake across time points (Figure 5O). HuMGEV-treated cells similarly exhibited stable vesicle internalization over time. Negative control cultures without sEV exposure showed no detectable CD63 signal, confirming the specificity of CD63-tdTomato-labeled vesicle uptake by HuSCs (Figure 5M,N). Overall, quantitative analyses demonstrated comparable uptake of mouse- and human-derived MGEVs across all time points, indicating similar uptake kinetics under the conditions tested (Figure 5O).

**Figure 6:**
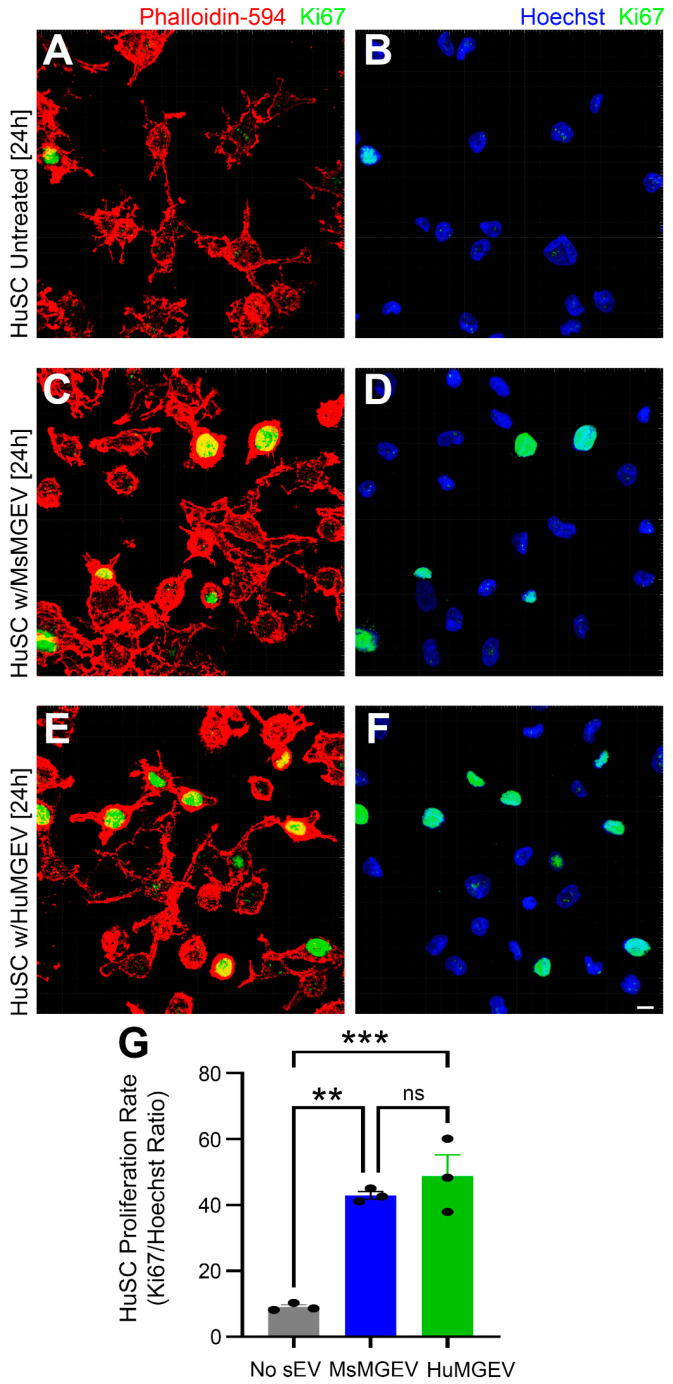
Effects of mouse- and human-microglia-derived sEVs on proliferation of HuSCs. (**A**–**F**) Confocal images of HuSC stained for F-actin (Alexa Fluor™ 594 Phalloidin, red) to mark the cell bodies in combination with the proliferation marker Ki67 (green), and nuclei (Hoechst, blue) were acquired at 24 h post-sEV treatment. (**A**,**B**) Untreated HuSC show low levels of Ki67 positivity, consistent with a predominantly low-proliferating, resting state. (**C**,**D**) HuSC exposed to MsMGEV sEVs exhibits a pronounced increase in Ki67-positive nuclei, indicating enhanced cell-cycle activity. (**E**,**F**) Treatment with HuMGEV similarly shows increased expression of Ki67 expression in HuSCs. (**G**) Quantification of HuSC proliferation based on Ki67/Hoechst ratio following 24 h of treatment with no sEVs, MsMGEVs, or HuMGEVs. Both MsMGEVs and HuMGEVs significantly increased HuSC proliferation relative to untreated controls (** *p* < 0.01, *** *p* < 0.001), with no significant difference (ns) between the sEV sources across species. Bars represent mean ± SEM; individual points represent biological replicates. Scale bar: 10 μm.

**Microglia-Derived sEVs Enhance Schwann Cell Proliferation.** To assess whether MGEVs affect HuSC proliferation, cultured HuSCs were treated with MsMGEVs or HuMGEVs immediately after plating and once cell adhesion was established. After 24 h, cells were fixed and stained for Ki67, a nuclear marker of proliferation. HuSCs that were not treated with MGEVs exhibited minimal Ki67 labeling (Figure 6A,B), consistent with a predominantly quiescent state. Both MsMGEV- and HuMGEV-treated HuSC displayed a substantial increase in Ki67-positive nuclei (Figure 6C,F), indicating sEV-mediated promotion of cell-cycle entry. Quantitative analysis of Ki67/Hoechst ratios demonstrated significant increases in proliferation following treatment with either MsMGEVs or HuMGEVs (** *p* < 0.01, *** *p* < 0.001) upon HuSC plate adherence, with no significant differences between species (Figure 6G). These results indicate that both mouse- and human-derived microglial sEVs exhibit pro-proliferative effects on HuSCs.

**Figure 7:**
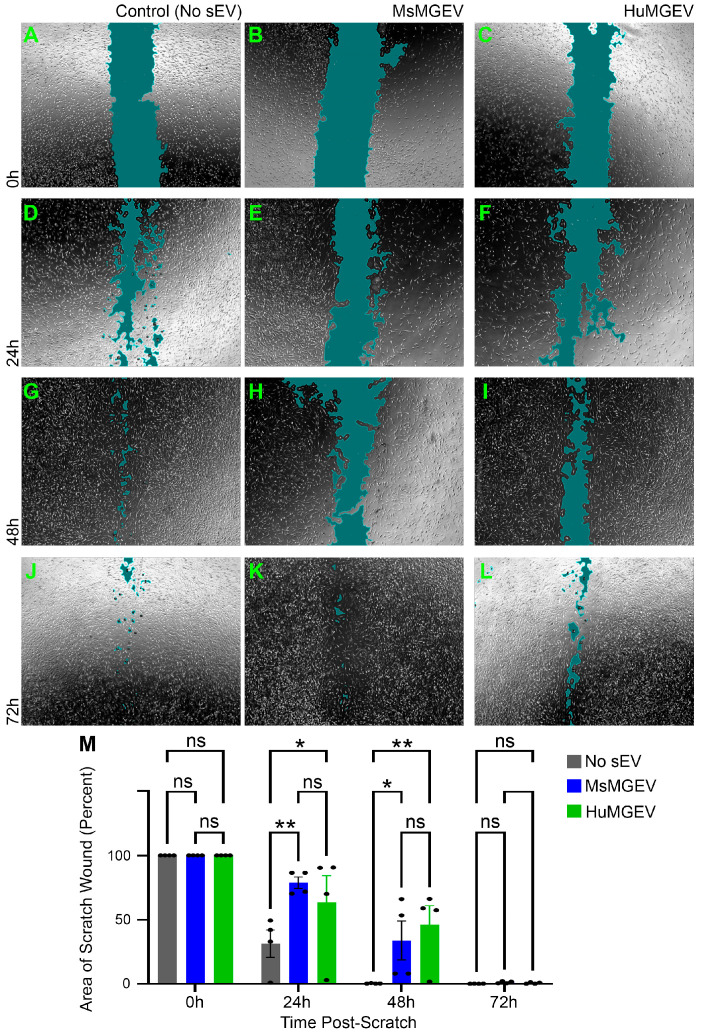
MGEVs modulate migration of HuSCs. (**A**–**L**) Representative phase-contrast images of HuSC monolayers subjected to a scratch wound assay and subsequently treated with no sEVs (**A**,**D**,**G**,**J**), MsMGEVs (**B**,**E**,**H**,**K**), or HuMGEVs (**C**,**F**,**I**,**L**). Images were captured at 0 h, 24 h, 48 h, and 72 h post-scratch. The turquoise-colored overlay marks the scratch wound area quantified at each time point. Relative to untreated controls, both MsMGEV- and HuMGEV-treated cultures show a reduction in the rate of wound closure, particularly evident at 24 h and 48 h. By 72 h, near-complete wound closure was observed across all conditions. (**M**) Quantitative evaluation of scratch wound area over time, expressed as a percentage of the initial wound area. At 24 h, MsMGEV treatment exhibited a slower rate of wound area reduction compared with untreated control (** *p* < 0.01) but showed no significant difference relative to HuMGEVs. By 48 h, both MsMGEVs (* *p* < 0.05) and HuMGEVs (** *p* < 0.01) continued to show reduced wound closure rates compared to the untreated control. No significant differences (ns) were detected between groups at 0 h or 72 h. Bars represent mean ± SEM; individual points represent biological replicates.

**Microglial sEVs Modulate Schwann Cell Migration in a Scratch Wound Assay.** Scratch wound assays were used to determine whether MGEVs regulate HuSC migration. Phase-contrast imaging showed that untreated HuSC monolayers gradually closed the wound area across 72 h (Figure 7A,D,G,J), whereas both MsMGEV- and HuMGEV-treated cultures exhibited delayed wound closure (Figure 7B,E,H,K and Figure 7C,F,I,L, respectively), particularly at early time points (24–48 h). Quantitative wound area measurements confirmed that MsMGEV treatment significantly slowed wound reduction at 24 h (** *p* < 0.01) and 48 h (* *p* < 0.05), while HuMGEVs similarly impaired wound closure at 48 h (** *p* < 0.01) (Figure 7M). By 72 h, all groups achieved near-complete wound closure with no remaining differences between treatments. These findings suggest that MGEVs transiently delay Schwann cell migration, with similar effects observed for both human- and mouse-derived sEV sources.

**Figure 8:**
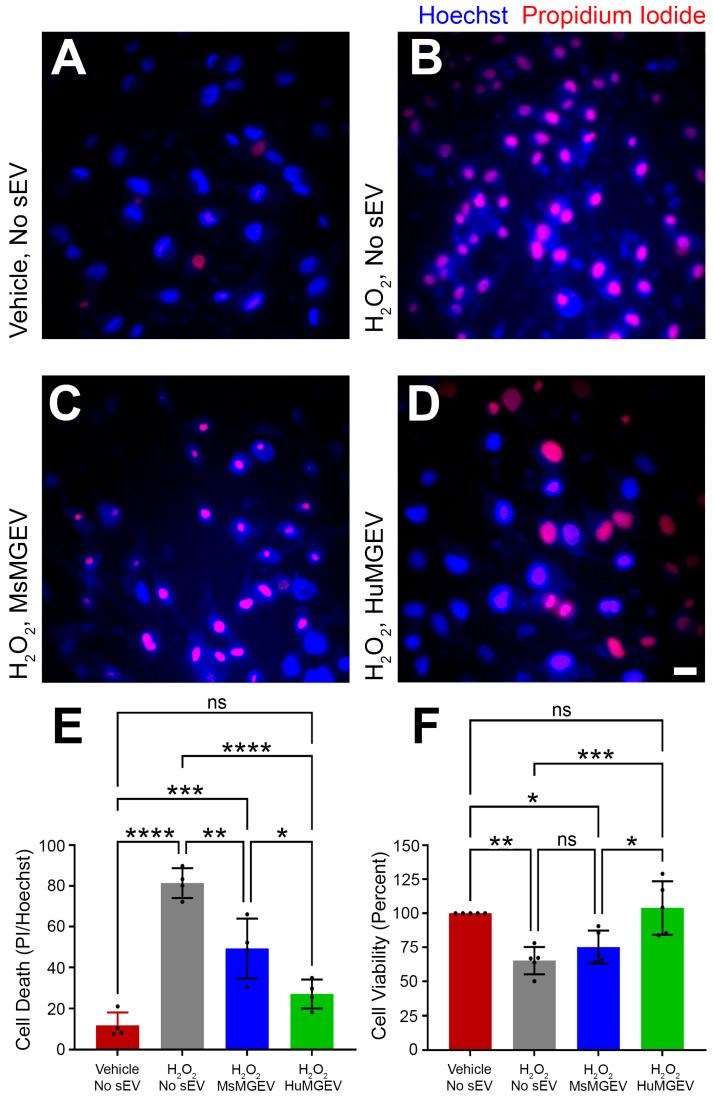
Human and mouse MGEVs protect HuSCs from oxidative stress-induced cell death. (**A**,**D**) Representative fluorescence images of HuSC exposed to vehicle or 50 μm hydrogen peroxide (H_2_O_2_) in the absence or presence of MGEVs of mouse or human origin. Nuclei were stained with Hoechst (blue) and non-viable cells with propidium iodide (PI; red). (**A**) Vehicle-treated (no H_2_O_2_) HuSC cultures show low PI staining, indicating high baseline viability. (**B**) H_2_O_2_ treatment markedly increases PI-positive cells, reflecting oxidative stress-induced cytotoxicity. (**C**,**D**) Co-treatment of HuSC cultures with H_2_O_2_ and MsMGEVs (**C**) or HuMGEVs (**D**) reduces PI staining compared with H_2_O_2_-exposure-alone group, demonstrating partial rescue from oxidative cytotoxicity. (**E**) Quantification of cell-death based on PI/Hoechst ratio. H_2_O_2_ treatment dramatically increased cell death (**** *p* < 0.0001), whereas both MsMGEVs (** *p* < 0.01) and HuMGEVs (**** *p* < 0.0001) significantly reduced PI-positive nuclei. HuMGEV treatment showed significantly increased protection than MsMGEVs (* *p* < 0.05). (**F**) Quantification of HuSC viability following H_2_O_2_ exposure alone or in combination with MGEVs of mouse or human origin expressed as percent of vehicle-treated controls. H_2_O_2_ significantly reduced cell viability compared to the untreated controls (** *p* < 0.01) as measured by CCK8 assay corresponding to the metabolic activity of living cells, while MsMGEV demonstrated a trend in increased viability but was statistically not significant (ns). HuMGEV treatments significantly improved survival relative to H_2_O_2_ alone (*** *p* < 0.001) or compared to H_2_O_2_ and MsMGEVs groups. Bars represent mean ± SEM; individual points represent biological replicates. Scale bar: 10 μm.

**Microglial sEVs Protect Schwann Cells from Oxidative Stress-Induced Cell Death.** To evaluate whether MGEVs confer cytoprotection, HuSC were exposed to hydrogen peroxide (H_2_O_2_) with or without sEV co-treatment. Compared to the control (vehicle treated, no H_2_O_2_, Figure 8A) which showed minimal cell death, H_2_O_2_ exposure markedly increased propidium iodide (PI)-positive nuclei (Figure 8B), indicating substantial cytotoxicity. Co-treatment with MsMGEVs or HuMGEVs significantly reduced PI staining (Figure 8C,D), demonstrating partial protection from oxidative damage. Quantification confirmed dramatic H_2_O_2_-induced cell death (**** *p* < 0.0001) and a significant rescue by both species’ MGEVs, though HuMGEVs conferred greater protection than MsMGEVs (* *p* < 0.05) (Figure 8E). CCK8 assays which evaluate cell viability and cytotoxicity by quantifying the metabolic activity of live cells, further showed that HuMGEVs significantly improved SC metabolism compared to H_2_O_2_ alone (*** *p* < 0.001), whereas MsMGEVs showed a non-significant trend toward improved viability (Figure 8F). These data indicate that MGEVs reduce oxidative stress-induced cytotoxicity in HuSCs, with human-derived MGEVs providing stronger cytoprotective effects. While the present study did not directly examine individual miRNAs in recipient Schwann cells, the observed functional effects are likely mediated by the coordinated and combinatorial action of multiple sEV-delivered miRNAs rather than by a single dominant miRNA species. This is consistent with the established paradigm of sEV-mediated signaling, in which vesicular cargo acts in an integrated and context-dependent manner together with other EV-associated components. Although transfection of individual miRNA mimics into HuSCs could provide mechanistic insight into specific regulatory contributions, such approaches may not fully recapitulate the complex and physiologically relevant signaling conveyed by intact MGEVs. Future studies employing targeted miRNA gain- and loss-of-function strategies in HuSCs will be important to delineate the relative contribution of specific conserved miRNAs identified here.

**Figure 9:**
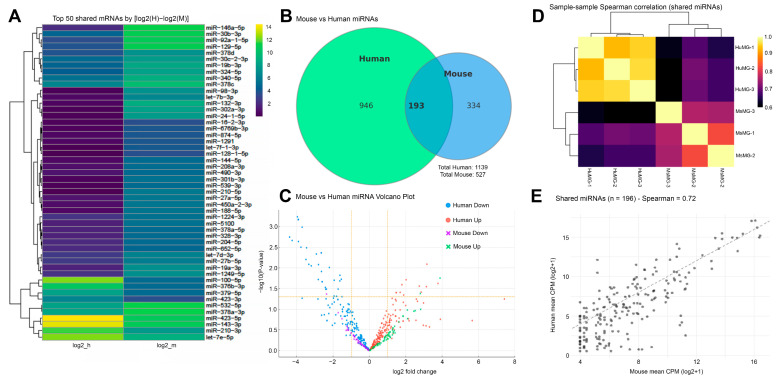
Comparative miRNA profiling of human- and mouse-microglia-derived small extracellular vesicles. (**A**) Heatmap of the top 50 shared miRNAs ranked based on the largest expression differences between human and mouse samples, ranked by |log_2_(Human CPM + 1) − log_2_(Mouse CPM + 1)|. Each row represents one miRNA, and each column represents the log_2_-normalized mean abundance in human and mouse sEV samples. Color intensity reflects standardized expression (row-scaled), revealing groups of miRNAs strongly enriched in one species. Hierarchical clustering highlights distinct expression patterns across species while confirming substantial overlaps in highly expressed vesicle-associated miRNAs. (**B**) Venn diagram illustrates the overlap of detectable miRNAs between human (*n* = 1139) and mouse (*n* = 527) MGEVs. A core set of 193 miRNAs is shared across species, whereas 946 miRNAs are unique to humans and 334 unique to mouse sEV preparations. (**C**) Volcano plot comparing differential miRNA expression between human and mouse MGEVs. Colored points denote significantly upregulated (red for human, green for mouse) and downregulated (blue for human, purple for mouse) miRNAs (cutoffs: |log_2_ fold change| ≥ 1 and −log_10_(*p*-value) ≥ 1). The majority of species-enriched miRNAs show modest fold changes, indicating broadly conserved miRNA expression profiles. (**D**) This panel shows a Spearman correlation heatmap generated using shared miRNAs identified across biological replicates of human and mouse sEV samples. High within-species clustering and strong cross-species correlations suggest conserved species-specific global miRNA signatures. Between-species correlations are lower, reflecting large-scale divergence in sEV-associated miRNA abundance. (**E**) Scatterplot comparing log_2_-normalized mean CPM of each shared miRNA between human versus mouse MGEVs. Each point represents one miRNA; the dashed 1:1 line indicates equal abundance. Most miRNAs fall away from the diagonal, indicating species-biased expression. A strong positive correlation (Spearman r = 0.72) indicates preservation of relative miRNA abundance patterns between species despite global expression differences.

**Global miRNA Profiling Reveals Conserved and Species-Specific sEV miRNA Signatures.** Comprehensive miRNA sequencing of MGEVs from mouse and human revealed both conserved and species-biased miRNA expression patterns. A heatmap of the 50 miRNAs with the largest interspecies differences showed clear clustering of mouse- and human-enriched miRNAs, despite a large, shared core of abundant miRNAs (Figure 9A). Venn diagram analysis revealed 193 miRNAs within MGEVs that were shared between the two species, with substantially more unique miRNAs detected in human (*n* = 946) than in mouse (*n* = 334) sEVs (Figure 9B). A volcano plot identified significantly upregulated miRNAs in each species, although most differentially expressed miRNAs exhibited modest fold changes, indicating broad conservation of sEV-associated miRNA profiles (Figure 9C). Spearman correlation heatmaps showed strong within-species clustering and moderate cross-species correlation, reflecting conserved global expression patterns yet distinct species-dependent abundance profiles (Figure 9D). Scatterplot comparison of shared miRNAs demonstrated a strong positive correlation (r = 0.72), highlighting overall preservation of miRNA abundance hierarchies despite apparent interspecies differences (Figure 9E). Scatterplot comparison of shared miRNAs demonstrated a strong positive correlation (r = 0.72), highlighting overall preservation of miRNA abundance hierarchies despite interspecies differences (Figure 9E).

**Figure 10:**
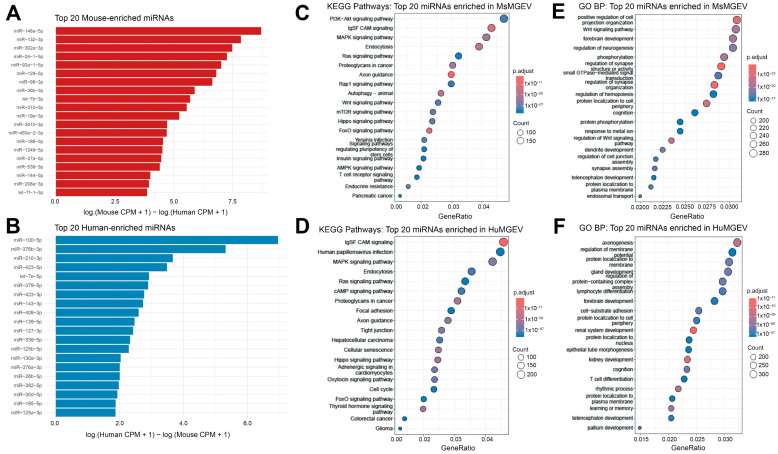
Identification of species-enriched miRNAs in microglia-derived sEVs and associated functional pathway analysis. (**A**) Top 20 mouse-enriched miRNAs. The bar plot highlights the top 20 miRNAs most enriched in mouse sEV samples, ranked by the abundance difference log2(Mouse CPM + 1) − log2(Human CPM + 1). These miRNAs represent mouse-dominant regulatory signatures and reflect species-specific biology underlying sEV function. (**B**). Top 20 human-enriched miRNAs. This bar plot shows the top 20 miRNAs enriched in human sEV samples using the inverse abundance difference log2(Human CPM + 1) − log2(Mouse CPM + 1). These miRNAs represent human-dominant sEV signatures and potentially target pathways with limited conservation in mouse. (**C**,**D**) KEGG pathway enrichment analysis for the top 20 mouse-enriched (**C**) and human-enriched (**D**) miRNAs. Pathways significantly associated with mouse MGEVs include PI3K-Akt signaling, MAPK signaling, Rap1 signaling, endocytosis, and axon guidance. Human-enriched miRNAs map to pathways such as MAPK signaling, proteoglycans in cancer, endocytosis, focal adhesion, Hippo signaling, and cell-cycle regulation. Dot size corresponds to the number of miRNAs associated with each pathway, and dot color reflects adjusted *p*-values. (**E**,**F**) Gene Ontology (GO) showing Biological Process (BP) enrichment for the top 20 mouse-enriched (**E**) and human-enriched (**F**) miRNAs. Mouse-associated processes include cell differentiation, Wnt signaling, synapse assembly, response to metal ions, endosomal transport, and dendrite development, highlighting roles in neuron–glia interaction and vesicular trafficking. Human-enriched GO terms include axonogenesis, membrane regulation, neurogenesis, immune-related processes, cognition, and forebrain development. Together, these analyses reveal both shared and species-specific biological functions potentially regulated by sEV miRNAs.

**Species-specific miRNA Signatures and Pathway Enrichment in Mouse and Human Microglia-derived sEVs.** To define conserved and species-restricted regulatory signatures in MGEVs, we compared miRNA expression profiles in resting mouse and human MGEVs. Differential abundance analysis revealed distinct sets of miRNAs selectively enriched in each species (Figure 10A,B). MsMGEVs displayed high levels of miR-144-5p, miR-142a-3p, miR-30 family members, and miR-129-5p, whereas HuMGEVs were enriched in miR-100-5p, miR-378 family members, miR-210-3p, and miR-423-3p. These differences in miRNA profiles between mouse and human sEVs suggest that microglia from each species within these in vitro models load distinct sets of miRNAs into the vesicles they release, although contributions from immortalization history and culture adaptation cannot be excluded. To determine the functional implications of these divergent miRNA repertoires, we performed KEGG pathway enrichment analysis using the top 20 enriched miRNAs from each species (Figure 10C,D). Mouse-enriched miRNAs were associated with signaling cascades central to microglial physiology, including PI3K-Akt, MAPK, Ras, mTOR, Hippo, and autophagy pathways, as well as had processes linked to neuronal communication such as axon guidance and synaptic regulation. Additional enrichment in insulin, AMPK, and T cell receptor pathways suggested potential cross-talk between metabolic and immune signaling mediated by MsMGEV miRNA cargo.

In contrast, human-enriched miRNAs mapped to a distinct yet partially overlapping set of KEGG pathways. HuMGEV signatures prominently involved IgG Fc/adhesion molecule interactions, tight junction regulation, cellular senescence, and pathways associated with oncogenic or proliferative signaling such as MAPK, Hippo, and cell-cycle control. Enrichment in adrenergic and oxytocin signaling, as well as multiple cancer-related pathways, indicated a unique spectrum of regulatory targets characteristic of human microglial vesicle biology.

Gene Ontology Biological Process (GO-BP) analysis further resolved species-specific distinctions (Figure 10E,F). Mouse-enriched miRNAs were associated with processes related to neuronal organization such as dendrite development, synapse assembly, and neurotransmission as well as autophagy, protein phosphorylation, endosomal transport, and regulation of small GTPase signaling. These pathways collectively pointed to strong engagement of MsMGEVs in maintaining neuronal connectivity and intracellular trafficking pathways.

Conversely, human-enriched miRNAs were linked to axonogenesis, membrane dynamics, and epithelial and kidney morphogenesis, alongside neurodevelopmental processes including cognition and neurogenesis.

Additional enrichment in proteolysis-related and substrate–ubiquitination pathways highlighted a differential emphasis on protein homeostasis in HuMGEVs compared to their murine counterparts.

Together, our findings show that resting mouse and human microglia release sEVs with distinct, species-specific miRNA cargo that while converging on shared neuroimmune pathways encode divergent regulatory landscapes. These species-specific miRNA enrichment patterns likely reflect evolutionary divergence in microglial function and suggest that MsMGEVs and HuMGEVs differentially shape the physiology of recipient cells.

**Figure 11:**
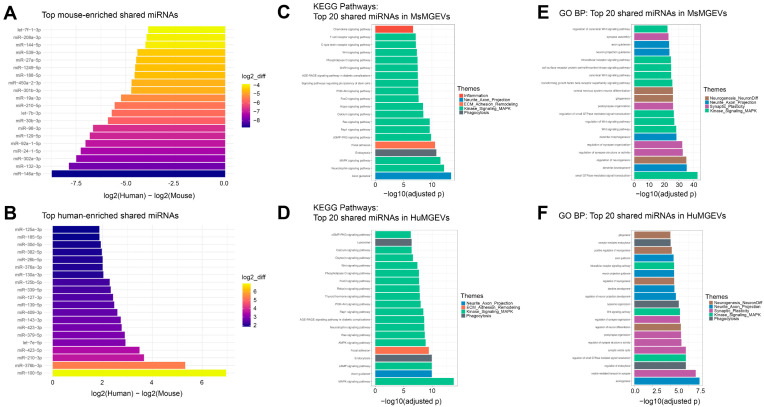
Functional enrichment analysis of shared miRNAs differentially expressed between mouse and human MGEVs indicating apparent species-biased enrichment. (**A**,**B**) Bar graphs showing the top differentially enriched *shared* miRNAs—those detected in both species but expressed at significantly different levels. (**A**) Top mouse-enriched shared miRNAs ranked by log_2_(Human) − log_2_(Mouse) abundance, with negative values indicating higher expression in mouse MGEVs. (**B**) Top human-enriched shared miRNAs, with positive log_2_ differences indicating higher abundance in HuMGEVs. Color gradients reflect magnitude of differential expression (log_2__diff). KEGG pathway enrichment analysis of the top 20 shared miRNAs in mouse (**C**) and human (**D**) microglial sEVs. Bars display −log_10_(adjusted *p*-values) for significantly enriched pathways. Pathways are grouped by microglia specific functional themes (color-coded), including inflammation, neurite/axon projection, ECM/adhesion/remodeling, kinase/MAPK signaling, and phagocytosis/vesicle-mediated processes. Both species show enrichment of multiple signaling cascades such as MAPK, PI3K-Akt, Ras/Rap1, with species-specific differences in the relative contribution of immune, endocrine, and metabolic pathways. (**E**,**F**) Gene Ontology Biological Process (GO-BP) enrichment analysis for the same species-enriched shared miRNA sets in MsMGEVs (**E**) and HuMGEVs (**F**). Top enriched GO terms are shown on a −log_10_(adjusted *p*) scale. Pathways cluster into neurodevelopmental processes, neuronal projection and differentiation, MAPK/kinase signaling modules, cell adhesion, and phagocytic/immune-regulatory functions.

Overall, the GO BP landscape in HuMGEVs highlights a conserved emphasis on neuronal development, axon projection, synaptic organization, and intracellular kinase signaling, but with a distribution distinct from that seen in MsMGEVs, indicating species-specific shifts in how shared miRNAs weight neurodevelopmental versus synaptic and signaling programs (Figure 11). Notably, several highly abundant miRNAs shared between mouse and human MGEVs, including members of the let-7 family, miR-132-3p, miR-146a-5p, miR-27a-5p, and the miR-30 family, have been previously implicated in the regulation of cell proliferation, migration, and resistance to oxidative stress. Consistent with these associations, pathway enrichment analyses of shared miRNAs identified key signaling pathways such as PI3K-AKT, MAPK, Hippo, and Wnt signaling. In the context of our experiments examining the effects of MGEVs on HuSC responses, these pathways are well-established regulators of Schwann cell biology. Collectively, these findings suggest that conserved miRNA cargo contributes at least in part to the pro-proliferative and cytoprotective effects of MGEVs observed in HuSCs. Table 1 summarizes the key signaling axes targeted by the top mouse-enriched MGEV miRNAs, highlighting their established roles in PI3K-AKT, MAPK/ERK, Wnt/β-catenin, TGF-β/Smad, Hippo-YAP/TEAD, NF-κB, and neuroimmune regulatory pathways as supported by prior studies listed in the table. Similarly, Table 2 summarizes the top 20 HuMGEV miRNAs shared with MsMGEVs and their established CNS functions, highlighting roles in neuroinflammation, glial activation, macrophage/microglial polarization, autophagy and apoptosis, cellular senescence, hypoxia responses, neuropathic pain, and mTOR/IGF1R- and PI3K-AKT-linked survival and metabolic pathways.

**Figure 12:**
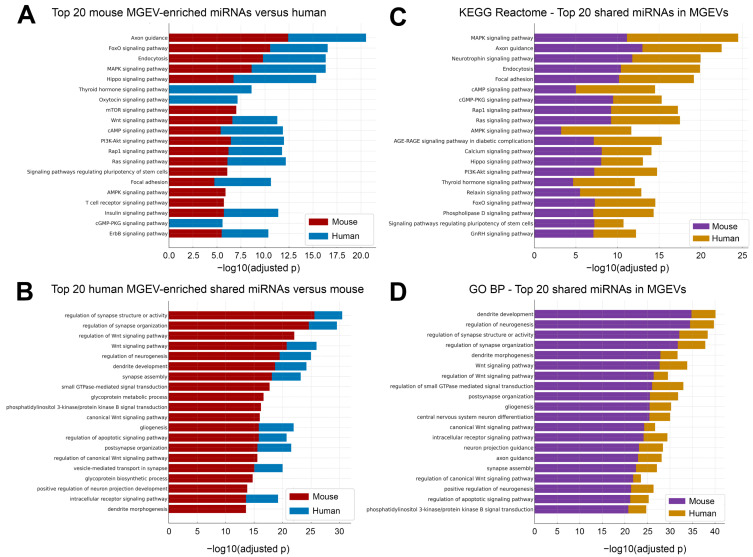
Comparative pathway enrichment analysis of shared miRNAs differentially expressed between mouse and human MGEVs. (**A**) KEGG pathway enrichment of the top mouse MGEV-enriched miRNAs compared to human MGEV mRNAs. Bars represent −log_10_(adjusted *p*-value) for pathways significantly associated with miRNAs more abundant in mouse MGEVs. Highly enriched pathways include axon guidance, FoxO signaling, endocytosis, MAPK signaling, Hippo signaling, and several hormone- and kinase-mediated signaling pathways, indicating strong associations with neuronal communication, intracellular trafficking, and cellular stress responses. (**B**) KEGG pathway enrichment of the top human-enriched miRNAs compared to mouse MGEV mRNAs. Human-enriched MGEV miRNAs mapped to pathways related to synapse structure and activity, Wnt signaling regulation, neurogenesis, dendrite development, small GTPase signaling, and gliogenesis. These processes emphasize human-specific enrichment in neurodevelopmental and synaptic regulatory pathways. (**C**) Cross-species KEGG Reactome comparison for the top 20 shared miRNAs between mouse and human MGEVs. Bars show −log_10_(adjusted *p*-value) for mouse (purple) and human (gold) enrichment. While numerous pathways like MAPK signaling, axon guidance, neurotrophin signaling, cAMP signaling, and Rap1 signaling are common, the degree of enrichment varies. This variation emphasizes how different species apparently contribute uniquely to important signaling cascades. (**D**) GO Biological Process (GO BP) enrichment analysis of the top 20 shared miRNAs in mouse (purple) and human (gold) MGEVs. Strong enrichment is observed in dendrite development, neurogenesis, synapse organization, Wnt signaling regulation, gliogenesis, intracellular receptor signaling, and axon guidance. Both species show overlapping GO themes, but human MGEVs display higher enrichment in pathways associated with synaptic function and neuronal differentiation, whereas mouse MGEVs show stronger enrichment in Wnt- and GTPase-mediated signaling.

**Comparative Cross-Species Profiling Reveals Distinct Signaling and Neurogenic Pathways Targeted by Microglial sEV miRNAs.** KEGG pathway enrichment of the top 20 mouse MGEV-enriched miRNAs to that of miRNA expression in HuMGEVs (Figure 12A) revealed significant association with a wide range of intracellular signaling cascades. The highest log_10_(adjusted *p*) values were observed for axon guidance, FoxO signaling, MAPK signaling, and endocytosis. Additional enriched pathways included Hippo signaling, thyroid hormone signaling, oxytocin signaling, mTOR signaling, Wnt signaling, cAMP signaling, PI3K-Akt, Rap1, and Ras signaling. Mouse-enriched miRNAs also mapped to immune-related pathways such as T cell receptor signaling and C-type lectin receptor signaling, as well as metabolic and growth-related pathways including insulin signaling, AMPK signaling, cGMP-PKG signaling, ErbB signaling, and signaling pathways regulating pluripotency of stem cells. Similarly, the top 20 human-enriched miRNAs compared to the miRNA profile of MsMGEV (Figure 12B) showed strong enrichment central to neuronal and synaptic function. The most significant categories included regulation of synapse structure or activity, regulation of synapse organization, and regulation of Wnt signaling pathway. Additional enriched processes involved regulation of neurogenesis, dendrite development, synapse assembly, gliogenesis, post-synapse organization, and dendrite morphogenesis. Human-enriched miRNAs also targeted intracellular trafficking and signaling pathways, including small GTPase-mediated signal transduction, vesicle-mediated transport in synapse, glycoprotein metabolic and biosynthetic processes, intracellular receptor signaling, and phosphatidylinositol 3-kinase/protein kinase B signaling.

Similarly, a side-by-side comparative expression of the top 20 shared miRNAs between HuMGEVs and MsMGEVs based on the KEGG pathway analysis demonstrated substantial overlap in pathway identity across the two species (Figure 12C). Both showed enrichment for MAPK signaling, axon guidance, neurotrophin signaling, endocytosis, focal adhesion, cAMP signaling, cGMP-PKG signaling, Rap1, Ras, AMPK, calcium signaling, Hippo signaling, PI3K-Akt signaling, thyroid hormone signaling, relaxin signaling, FoxO signaling, phospholipase D signaling, pluripotency regulation, and GnRH signaling. Despite the shared pathway set, bar heights indicated greater enrichment in human for several pathways, most notably MAPK, axon guidance, neurotrophin signaling, cGMP-PKG, and Rap1, while a smaller number of pathways showed relatively higher enrichment in mouse. The same top 20 shared miRNAs between mouse and human sEVs subjected to GO BP analysis revealed conserved enrichment for major neurodevelopmental and synaptic processes in both species (Figure 12D). Key categories included dendrite development, regulation of neurogenesis, regulation of synapse structure or activity, regulation of synapse organization, dendrite morphogenesis, Wnt signaling regulation, postsynaptic organization, gliogenesis, CNS neuron differentiation, intracellular receptor signaling, axon guidance, synapse assembly, positive regulation of neurogenesis, and PI3K-Akt-related signaling. Although these categories were common to both mouse and human, the enrichment values were generally higher in human, indicating species-specific differences in how shared miRNAs influence neurodevelopmental and synaptic gene networks.

## 4. Discussion

In this study, we present the first comprehensive, multimodal cross-species analysis of microglia-derived sEVs, integrating their production, isolation, and characterization from well-established mouse (BV2) and human (HMC3) microglial cell lines under matched culture conditions and systematically evaluating their bioactivity in HuSCs. To minimize technical variability, all experimental procedures including cell culture, sEV purification, particle characterization, functional assays, and miRNA sequencing were performed using identical protocols, reagents, and analysis pipelines across species. Where species-specific reagents were required, validated human- or mouse-specific antibodies were used, with quantitative analyses conducted within species and normalized accordingly. miRNA profiling was likewise performed using the same library preparation, sequencing platform, and bioinformatic workflow for both species. Although minor species-dependent differences in detection efficiency cannot be fully excluded, the consistency of findings across multiple independent readouts including biophysical, molecular, functional, and miRNA-based analyses supports the conclusion that the observed differences predominantly reflect biological rather than technical variation. Although our isolation strategy differs from classical ultracentrifugation-based workflows, the SEC-centered approach used here is a rapid and scalable method well suited for large-scale studies, providing improved size-based purity and reproducibility while better preserving sEV structural and functional integrity by avoiding harsh centrifugation as described previously [44]. When combined with stringent marker-based validation, SEC effectively removes most soluble protein contaminants and lipoproteins that commonly co-isolate with sEVs during ultracentrifugation, thereby enhancing sample purity. However, occasional late-fraction particle peaks observed in SEC workflows, such as those detected for HuMGEV fraction 9, were consistent with non-vesicular components and were therefore stringently excluded from all subsequent downstream analyses. By integrating biophysical, molecular, functional, and miRNA profiling, we reveal that mouse and human MGEVs share a conserved core of neuroprotective functions yet diverge markedly in vesicle size, tetraspanin fingerprints, basal vesicle load, and vesicular miRNA cargo as summarized in Table 3. These species-specific differences reshape regenerative and cytoprotective responses in human recipient cells and expose fundamental divergence in sEV biology across species. While these differences are consistent with known species-level divergence in microglial biology, we cannot exclude the possibility that immortalization history and cell line-specific regulatory programs contribute to the observed sEV phenotypes. Together, our findings both delineate the strengths and determine the limitations of murine models for studying human MGEVs, refining the interpretation of rodent MGEV data in human disease contexts and providing new insights into an apparent species divergence in sEV biology and a rational blueprint for the design of human-relevant, sEV-based therapeutic strategies. These findings refine the translational relevance of rodent sEV data and provide a critical foundation for designing sEV-based therapeutics that more accurately reflect human-specific biology.

**Species-Dependent sEV Biogenesis And Biochemical Composition.** Our initial characterization demonstrated that both MsMGEVs and HuMGEVs segregated into early SEC fractions and expressed canonical sEV markers, consistent with MISEV2024 guidelines [12]. Both preparations were enriched for TSG101 and depleted of calnexin, confirming successful isolation of vesicle-enriched fractions with minimal non-vesicular contamination. However, NTA revealed significantly larger mean and mode diameters for MsMGEVs relative to HuMGEVs, despite overlapping size ranges by TEM. Prior work indicates that sEV size is influenced by endosomal dynamics, membrane curvature, and activation state [108], suggesting that microglial vesicle biogenesis may be tuned differently in mouse versus human cells. These biophysical differences are not merely technical details; they have translational consequences. Vesicle size affects biodistribution, uptake route, and clearance in vivo, with smaller EVs often penetrating tissue barriers and larger vesicles engaging distinct endocytic pathways. Thus, sEV formulations optimized in mice may not reproduce the same pharmacokinetic or targeting profiles in humans if underlying size distributions are intrinsically species-biased. Incorporating species-specific size signatures into sEV design and dosing strategies may therefore be necessary to improve translation from preclinical to clinical settings.

**Tetraspanin Expression Profiling Revealed Species-Specific Molecular Fingerprints.** Quantitative profiling of canonical sEV tetraspanins revealed distinct molecular fingerprints between MsMGEVs and HuMGEVs. MsMGEVs were enriched in CD9, whereas HuMGEVs showed higher CD81 expression, while CD63 levels and TSG101 abundance were largely comparable. Tetraspanins are central organizers of membrane microdomains and not only regulate membrane organization, but also influence cargo sorting, interaction with target recipient cells, and sEV biodistribution [109,110]. Our donut plots clearly highlight how species divergence dictates the proportional distribution of these markers, suggesting caution when interpreting tetraspanin “signatures” across species. From a translational perspective, this has two major implications. First, it suggests that tetraspanin-centric strategies for sEV capture, enrichment, or targeting (such as CD9- or CD81-based isolation, antibody-decorated scaffolds) may preferentially enrich distinct sEV subpopulations in mouse versus human samples, potentially biasing readouts. Second, species-dependent tetraspanin patterns may contribute to differences in how MGEVs engage neural and non-neural targets in vivo. Future work that functionally links these tetraspanin profiles to specific uptake routes or receptor interactions could inform rational engineering of sEVs for targeted delivery in human therapies.

**Intrinsic Species Differences In Microglial sEV Abundance.** We also observed significantly greater CD63-positive intracellular vesicle load in BV2 mouse microglia relative to human HMC3 microglia. This finding aligns with previous studies conducting high-resolution quantitative proteomics of human and mouse microglia that revealed a shared core proteome but pronounced species- and culture-dependent differences in inflammatory, as well as basal metabolic and phagocytic activity, and disease-associated proteins [27,111], which are expected to drive corresponding alterations in the molecular composition and function of sEVs they generate. Such differences may underlie some of the differences in vesicular signaling activity relative to human microglia, a consideration critical for translational modeling. This suggests that mouse microglia may likely exaggerate the basal vesicular “activity” of microglial signaling relative to human microglia, particularly in resting or low-activation states. This is especially relevant for disease models where MGEVs are used as biomarkers or therapeutic agents: differences in baseline vesicle production rates could distort attempts to extrapolate sEV dose, timing, or functional thresholds from rodent data to human patients. However, because our imaging approach does not explicitly distinguish multi-vesicular bodies (MVB) from other CD63-positive endosomal compartments, definitive attribution of this increased vesicle load to MVB activity will require ultrastructural analysis or co-localization with additional endosomal markers in future studies.

**Conserved Schwann Cell Uptake but Species-Biased Functional Responses.** Both MsMGEVs and HuMGEVs were readily internalized by HuSC in a time-dependent manner, consistent with known vesicle uptake mechanisms involving actin remodeling and endocytosis [112]. Interestingly, while qualitative imaging suggested subtle differences in vesicle accumulation patterns, statistical analysis of uptake revealed no significant differences between mouse and human MGEVs, indicating broadly similar internalization behavior in HuSC. Although uptake per se was not species restricted, the downstream consequences on Schwann cell behavior revealed important divergences.

Functionally, both mouse- and human-derived MGEVs enhanced Ki67-driven HuSC proliferation, shifting newly plated cells from a predominantly quiescent state into a more proliferative phenotype, while transiently slowing migration in wound scratch assays. This reciprocal regulation of proliferation and migration is consistent with previous observations that cells often trade off migratory capacity when engaging cell-cycle programs [113]. In a regenerative context, when examining the transplantation of SCs into the injured spinal cord, these findings suggest that MGEVs may support expansion of Schwann cell populations at early stages of repair while temporarily tempering migration, potentially influencing timing and patterning of remyelination or reinnervation.

From a translational angle, the fact that both mouse- and human-derived MGEVs drive broadly similar proliferative and migratory shifts in HuSC is encouraging as it supports the existence of a conserved pro-regenerative sEV “core function” that can be interrogated in rodent models. However, because we found species-specific differences in miRNA cargo and differences in how strong the functional effects were, MsMGEVs may not reliably substitute for HuMGEVs when predicting their cryoprotective or regenerative potential.

**Human MGEVs Provide Greater Protection Against Oxidative Stress.** A key translationally relevant observation was that both MsMGEVs and HuMGEVs mitigated H_2_O_2_-induced cytotoxicity in HuSCs, but HuMGEVs conferred significantly more robust protection at both the cell death and viability levels. This suggests that while MGEVs from both species carry cytoprotective programs, the human vesicle cargo is more efficient in buffering oxidative stress in human cells. However, the specific outcome of MGEV-mediated modulation of oxidative stress is highly context-dependent, and available literature has not established if human cells respond more robustly to human MGEVs than to mouse MGEVs for oxidative stress modulation. Differences in the molecular cargo of the MGEVs, including specific miRNAs and proteins between human and mouse cells, likely lead to potentially different functional outcomes in recipient cells. The stronger rescue by HuMGEVs may reflect differences in miRNA content, antioxidant pathway regulation, or vesicle surface signaling molecules.

Clinically, oxidative stress is a central pathology across CNS and PNS injuries and neurodegenerative diseases. Our findings imply that HuMGEVs or MsMGEV formulations engineered to mimic their miRNA/protein signatures may offer superior translational potential for therapeutic applications targeting oxidative damage. At the same time, the measurable but weaker benefit of MsMGEVs on human cells helps explain why some rodent sEV-based interventions show strong preclinical benefit yet may underperform when conceptually translated into human systems. Optimizing sEV cargo toward human-enriched cytoprotective miRNAs and pathways identified here could bridge that gap. These findings therefore underline the translational relevance of HuMGEVs when modeling neuroprotection.

**miRNA Profiling Reveals a Conserved Core With Marked Species-Specific Regulatory Bias.** High-resolution miRNA profiling revealed extensive overlap in MGEV-associated miRNAs, with a shared core of 193 vesicle-associated miRNAs between species consistent with the conserved role of microglia in neuronal support and immune surveillance [114], along with a substantially larger set of human-unique miRNAs relative to mouse. This aligns with broader evidence that human microglia exhibit greater regulatory complexity and distinct transcriptional programs relative to mouse microglia [115,116]. The strong positive correlation in rank-order expression of shared miRNAs (Spearman r = 0.72) suggests that MGEVs across species preserve a conserved hierarchy of regulatory signals, likely reflecting fundamental roles in homeostasis, synapse modulation, and immune surveillance [114]. At the same time, numerous shared miRNAs exhibited pronounced species-biased abundance, and many were species-restricted as evident from volcano plots and heatmaps that clearly indicated species-biased abundance among many shared miRNAs, highlighting evolutionary divergence in microglial regulatory pathways. This combination-conserved repertoire but divergent weighting has direct translational implications. Although rodent models may accurately capture which pathways MGEVs can modulate but not necessarily the types and the magnitude with which those pathways are activated in humans. This distinction is crucial when prioritizing miRNA candidates for biomarker discovery or therapeutic modulation: miRNAs that appear modestly enriched in MsMGEVs may be strongly upregulated in HuMGEVs (or vice versa), altering their relevance for clinical translation.

**Pathway Enrichment Reveals Both Shared Microglial Functions and Species-Specific Regulatory Programs.** Human microglia, when compared to those from rodents, consistently show a wider and more diverse expression of genes involved in neuronal development and synapse formation. Differences in the expression patterns of certain microRNAs, especially those unique to primates, are believed to play a major role in this divergence, contributing to the greater complexity of neural processing seen in the human brain. Pathway enrichment analyses demonstrated that mouse-enriched miRNAs were associated with PI3K-Akt, MAPK, Rap1, and axon guidance pathways, all consistent with microglial roles in cytoskeletal remodeling, phagocytosis, and neuronal interaction [117,118,119]. On the other hand, HuMGEV-enriched miRNAs mapped more strongly to neurogenesis, dendrite development, synapse organization, focal adhesion, and GTPase-mediated signaling, processes that reinforce the emerging view that human microglia are particularly tuned to modulate circuit-level plasticity and higher-order neural functions [117,120,121].

Importantly, several fundamental pathways such as MAPK signaling, axon guidance, ECM remodeling, and cAMP signaling were shared between both species, but the magnitude and ranking of enrichment differed, revealing functional divergence despite conserved pathway presence. Translationally, this suggests that rodent MGEV studies are likely valid for identifying qualitative pathway engagement but may misestimate the *relative* importance of specific signaling axes in human disease. Recognizing these species-dependent biases can guide more rational selection of targets for sEV engineering, focusing, for example, on human-enriched miRNAs and pathways when designing sEV-based therapies for synaptic stabilization, neuroprotection, or cognitive recovery.

**Innovation and Translational Significance.** This study offers several advances with direct translational relevance. Foremost, it establishes the first integrated cross-species EV framework under matched conditions by standardizing culture, isolation, and characterization procedures for BV2 and HMC3 microglia and assessing their effects in a common HuSC model, thereby isolating species as the primary variable and providing a template that can be extended to other glial and neuronal cell types. Second, it identifies species-specific tetraspanin and vesicle signatures as critical translational variables, demonstrating that commonly used sEV “identity markers” are not species agnostic and cautioning against direct transfer of tetraspanin-based capture or diagnostic strategies from rodent systems to human biofluids. Third, it shows conserved yet quantitatively divergent regenerative and cytoprotective functions, as both MsMGEVs and HuMGEVs enhance HuSC proliferation and mitigate oxidative stress, but human MGEVs exert more potent protective effects, emphasizing the necessity of validating sEV actions in human cells or humanized models when selecting therapeutic candidates. Finally, by mapping cross-species miRNA landscapes, the study explains how rodent sEV data can be simultaneously informative and misleading, providing a mechanistic basis and practical roadmap for prioritizing human-enriched miRNAs and pathways for clinical translation. Collectively, these advances help reframe microglial EV research from a purely descriptive, preclinical field into one with a more explicit translational trajectory, bridging the gap between rodent models and human therapeutic design.

**Limitations and Model Considerations.** A key limitation of this study is the reliance on a single immortalized mouse microglial cell line (BV2) and a single immortalized human microglial cell line (HMC3) rather than primary microglia, which may not fully capture the phenotypic and functional heterogeneity of microglia across species. While both these models enable controlled, reproducible, and scalable sEV production required for direct cross-species comparison, immortalization and prolonged in vitro culture can alter transcriptional programs, vesicle biogenesis pathways, and cargo-loading mechanisms. Consequently, some differences observed between mouse and human MGEVs may reflect model- or cell line-specific features rather than purely species-intrinsic biology. We therefore interpret our findings as defining conserved and divergent sEV signatures within standardized microglial model systems, rather than as definitive representations of in vivo species differences.

An additional consideration is that sEV behavior characterized in vitro may differ substantially from MGEV signaling in vivo, where vesicle composition and function are dynamically shaped by tissue microenvironment, regional heterogeneity, cell–cell interactions, and inflammatory state. As such, functional effects observed under simplified in vitro conditions may not fully recapitulate in vivo MGEV activity. The translational relevance of the present study therefore lies in identifying conserved versus species-biased trends that inform interpretation of rodent sEV data in human-relevant contexts, rather than in making direct predictions of human in vivo responses or therapeutic efficacy. Similar to the use of immortalized microglial cell lines, this study employed an immortalized human Schwann cell line rather than primary Schwann cell cultures, which may limit the extent to which the observed responses fully reflect the phenotypic diversity and functional states of primary Schwann cells in vivo.

From a molecular profiling perspective, although miRNA sequencing provided insight into conserved and species-biased regulatory signatures within MGEVs, we acknowledge that species-specific sequence composition and adapter ligation efficiencies may still contribute to subtle biases in small RNA library representation. To mitigate this, all samples were processed using identical library preparation protocols and bioinformatic pipelines, and cross-species comparisons were restricted to conserved, shared mature miRNAs with identical miRBase annotations. Nonetheless, complementary validation approaches such as targeted RT-qPCR of selected miRNAs or the inclusion of synthetic spike-in controls would further strengthen cross-species quantification and are important directions for subsequent future studies.

From a mechanistic perspective, although miRNA profiling provided insight into pathways potentially underlying functional differences between mouse- and human-derived MGEVs, individual miRNA contributions were not directly tested using gain- or loss-of-function approaches in recipient Schwann cells. Moreover, we did not conduct integrated multi-omics analyses, such as proteomic or lipidomic profiling of MGEVs in this study. While proteomic analyses can yield important mechanistic information, cross-species protein-level comparisons are often complicated by species-specific expression patterns, post-translational modifications, and annotation biases. We therefore prioritized miRNA profiling, which represents a conserved and functionally active class of vesicular cargo well suited for comparative mouse-to-human analyses. Future studies integrating RNA, proteomic, and lipidomic profiling across multiple microglial models and in vivo systems will be essential to fully resolve cargo-specific mechanisms and strengthen translational relevance. Future studies incorporating multiple primary and induced microglial models, in vivo validation, and integrative multi-omics approaches will therefore be necessary to establish the generalizability, mechanisms, and translational relevance of MGEV signaling.

Taken together, our data show that mouse and human microglial cell lines release sEVs with conserved core functional effects on HuSCs, alongside distinct molecular signatures. Importantly, the species-associated differences reported here are supported by concordant results from multiple orthogonal approaches, including vesicle size profiling, ultrastructural analysis, membrane tetraspanin characterization, functional assays, and miRNA sequencing, reducing the likelihood that antibody affinity alone accounts for the observed differences. These differences are consistent with species-specific functional biases such as pro-regenerative versus pro-functional/homeostatic signaling rather than absolute differences in activity. Although MGEV effects are influenced by recipient cell context, the persistence of these differences in a shared human Schwann cell system indicates a dominant contribution from donor-derived, species-specific properties. We interpret these findings as reflecting a combination of species bias and model-specific features inherent to immortalized microglia. Future studies using primary microglia or induced pluripotent stem cell-derived human microglia will be important to determine how broadly these sEV signatures extend to in vivo settings.

## 5. Conclusions

This study offers a systematic cross-species comparison of microglia-derived sEVs using standardized immortalized models, uncovering conserved functional effects alongside species-specific molecular signatures and highlighting key translational gaps that require validation in primary human and mouse microglia. We demonstrate that mouse and human MGEVs share conserved structural features, canonical vesicle markers, and cytoprotective functions, yet differ in vesicle size, tetraspanin composition, basal vesicle load, and miRNA cargo—differences that modulate their regenerative and protective effects in HuSCs. These findings indicate that murine EV models remain valuable for elucidating fundamental mechanisms but must be interpreted with explicit consideration of species-specific regulatory biases.

Integrated cross-species miRNA and pathway analyses further reveal that shared miRNAs are not functionally equivalent between species. Instead, species-biased enrichment patterns give rise to distinct regulatory architectures that diverge at the molecular level while converging on core neuroimmune and neuronal pathways. Together, these data establish that MGEV miRNA function is inherently species-context dependent and reflects evolutionarily tuned differences in microglial regulation that are critical when extrapolating between mouse and human systems.

Beyond defining these translational constraints, this work offers practical guidance for selecting appropriate preclinical models, informing the engineering of sEVs to emulate human-enriched protective signatures, and prioritizing miRNAs and pathways for biomarker and therapeutic development. Collectively, these findings support a more rational and biologically informed translation of microglial EV biology into human-relevant strategies for CNS and PNS injury and disease.

## Figures and Tables

**Table 1:** Top 20 MsMGEV miRNA shared with huMGEV and their associated functions in the CNS.

miRNA (MsMGEV)	Key Signaling Axis/Main Functional Targets
let-7f-1-3p	Regulation of neural stem cell differentiation [48], protection against oxidative [49] damage and maintenance of the blood–brain barrier integrity [50].
miR-208a-3p	No known primary function in the CNS. Primary role identified in cardiac function [cardiac remodeling, mitochondrial function, and electrical activity] [51].
miR-144-5p	Regulates mood, stress, and neurogenesis. Functions by negatively regulating inflammatory and apoptotic pathways, including the targets PTEN and TLR4, and by influencing neuronal processes like neurogenesis, synaptic plasticity, and inflammation [52].
miR-539-3p	Regulates hippocampal function, endothelial cell permeability, and neuroinflammation, primarily through its interaction with target genes like Lrp6 and SNAI2. It is also linked to the pathogenesis of stroke, where it can affect the blood–brain barrier, and plays a role in controlling inflammation in neurons [53,54].
miR-27a-5p	Context-dependent CNS roles, inhibiting oligodendrocyte maturation and remyelination, modulating neuroinflammation in microglia, and sometimes promoting or protecting against cell death [55,56].
miR-1249-5p	Specific role in the CNS is not well-defined. Associated with the inflammatory response via NF-κB signaling [57].
miR-188-5p	Regulates dendritic plasticity and synaptic transmission by downregulating Neuropilin-2 (Nrp-2). Restoring miR-188-5p levels can help recover synaptic and cognitive deficits linked to Alzheimer’s-related pathology [58]. Enhances cell survival and shown to inhibit apoptosis of neuronal cells during oxygen–glucose deprivation (OGD)-induced stroke by suppressing PTEN [59].
miR-450a-2-3p	Dysregulation associated with major depressive disorder (MDD) [60].
miR-301b-3p	Regulates inflammation and cognitive function and is linked to depression, where it can worsen hippocampal inflammation and cognitive deficits by activating NF-κB signaling, increasing microglial activation and pro-inflammatory cytokines such as TNF-α and IL-1β [61].
miR-19a-3p	Has context-dependent CNS effects, driving inflammation in ischemia and neuropathic pain after spinal cord injury, yet also supporting axonal growth and proliferation, with its inhibition reducing stroke injury [62]. May promote microglia activation and trigger inflammatory responses under pathological condition [63].
miR-210-5p	Regulates metabolic and inflammatory processes in the CNS, influencing neurogenesis, blood–brain barrier integrity, and repair in a hypoxia-sensitive, context-dependent manner that impacts mitochondria, astrocytes, and neural stem cell differentiation [64].
let-7b-3p	Key regulator of neural stem cell (NSC) proliferation and differentiation in the CNS by targeting nuclear receptor TLX signaling [65,66]. Increased expression of let-7b has been linked to neurodegenerative diseases through the activation of TLR7 signaling and associated inflammatory responses [67].
miR-30b-3p	Attenuates neuropathic pain by downregulating the voltage-gated sodium channel Nav1.3. [68] Promotes spinal cord sensory function recovery via the Sema3A/NRP-1/PlexinA1/RhoA/ROCK pathway [69].
miR-98-3p	Role in synaptic plasticity, learning, and memory [70]. Provides neuroprotective effect in response to acute injuries. Reduces inflammatory response [71].
miR-129-5p	Regulates neuronal development, synaptic plasticity, and neuroinflammation [66]. Essential for healthy astrocyte function by controlling glutamate uptake and preventing neuroinflammation [72].
miR-92a-1-5p	Promotes CNS autoimmunity by modulating the regulatory and inflammatory T cell balance [73]. May protect neurons against inflammatory neurodegeneration [74].
miR-24-1-5p	Not well-studied miRNA in the CNS.
miR-302a-3p	Supports nerve repair and neuroprotection by suppressing inflammation and enhancing cell survival and proliferation through inhibition of NF-κB signaling and downregulation of damage- and stress-related genes [75,76].
miR-132-3p	Regulates neuronal development, synaptic plasticity, and neuroprotection as well as promotes axon growth, nerve migration, and memory formation [77,78].
miR-146a-5p	Regulates inflammation by targeting inflammatory pathways like IRAK1/TRAF6 [79].

**Table 2:** Top 20 HuMGEV miRNA shared with MsMGEV and their associated functions in the CNS.

miRNA (HuMGEV)	Key Signaling Axis/Main Functional Targets
miR-125a-3p	Regulates oligodendroglial maturation [80]. Regulates human brain endothelial cell-barrier function in inflammation [81].
miR-185-5p	Regulates neural stem/progenitor cell growth and differentiation, supports neuronal survival and axon regeneration, and shown to alleviate neuropathic pain [82,83].
miR-30d-5p	Regulates autophagy and apoptosis in the CNS after injury [84].
miR-382-5p	Linked to increased microglial and astrocyte-induced inflammation and neuronal damage [85].
miR-26b-5p	Regulates neural differentiation-associated microRNAs and mRNAs by directly targeting REST [86]. Suppresses microglial activation and pro-inflammatory factors like IL-6 [87].
miR-376a-3p	Alleviates the development of glioma through negatively regulating KLF15 [88].
miR-130a-3p	Regulates neuronal morphology and differentiation by targeting Acsl4 [89].
miR-125b-5p	Modulates the function of regulatory T cells in tumor microenvironment by targeting TNFR2 [90].
miR-339-5p	Reduces inflammation by targeting inflammatory molecules like HMGB1 and the NF-κB pathway [91]. Inhibits alcohol-induced brain inflammation through regulating NF-κB pathway [90].
miR-127-3p	Has been shown to inhibit neurite outgrowth, induces cell apoptosis, and contributes to physiological dysfunction after spinal cord transection [92].
miR-139-5p	Improves functional recovery and reduces pain hypersensitivity after SCI [93].
miR-409-3p	Has been shown to promote microglial migration, activation and neuroinflammation by targeting Nr4a2 to activate the NF-κB pathway [94].
miR-143-3p	Modulates neuronal survival and function by targeting neuregulin-1 [95].
miR-423-3p	Plays a role in learning and memory, where it helps alleviate sevoflurane-induced dysfunction [96].
miR-379-5p	Alleviates neuronal injury following conditions like cerebral ischemia and spinal cord injury. Reduces autophagy, regulates cell death, and improves locomotor function by targeting specific genes like MAP3K2 and influencing pathways such as JNK/c-Jun signaling [97,98].
let-7e-5p	Highly expressed in the central nervous system (CNS) and plays a role in stress resilience by regulating signaling pathways like PI3K-Akt and MAPK in the prefrontal cortex [99,100].
miR-423-5p	Confers neuroprotection by targeting the NLRP3 inflammasome signaling pathway [101].
miR-210-3p	Promotes axon regeneration, regulates autophagy, and affects cell survival under hypoxic conditions [102]. Has been shown to regulate the metabolic and inflammatory status of primary human astrocytes [103]. Has been shown to regulate autophagy through the AMPK/mTOR signaling pathway, reduces neuronal cell death and inflammatory responses, and enhances functional recovery following cerebral hemorrhage [104].
miR-376b-3p	Can regulate the function of GABAergic signaling, which is crucial for inhibiting nerve activity in the brain [105].
miR-100-5p	Has a dual role in the CNS, where it can both aggravate and alleviate damage, primarily by affecting neuroinflammation and cell survival [106,107].

**Table 3:** Key species-specific differences between MsMGEVs and HuMGEVs.

Category	MsMGEVs (Mouse)	HuMGEVs (Human)
Particle properties	Larger mean/mode diameters; single sharp SEC peak (fraction 3).	Smaller particle diameters; broader SEC elution (fractions 2–4).
Tetraspanin profile	CD9-high, CD81-low.	CD81-high, CD9-low.
Resting microglia vesicle load	Higher intracellular CD63^+^ vesicle abundance.	Lower intracellular vesicle load.
Uptake by Schwann cells	Time-dependent accumulation (24–72 h).	Stable uptake across time (24–72 h).
Functional effects on Schwann cells	Equal proliferation boost; transient migration delay; moderate oxidative stress protection.	Equal proliferation boost; transient migration delay; stronger oxidative stress protection.
miRNA content	527 total miRNAs; 334 mouse-unique.	1139 total miRNAs; 946 human-unique.
Pathway bias	PI3K-Akt, MAPK, Rap1, endocytosis, Wnt/GTPase signaling.	MAPK, Hippo, focal adhesion, neurogenesis, synaptic and dendritic pathways.

## Data Availability

The data from the current study are available from the corresponding author upon reasonable request.
